# Hydraulic Pressure‐Programmed Molecular Transport in Tough Hydrogels

**DOI:** 10.1002/advs.77159

**Published:** 2026-08-20

**Authors:** Yijie Cheng, Shahd Alnasser, Akhiri Zannat, Zhenda He, Jiayi Zeng, Lenan Zhang, Sungmin Hong, Xinyue Liu

**Affiliations:** ^1^ Department of Chemical Engineering and Materials Science Michigan State University East Lansing Michigan USA; ^2^ Department of Mechanical Engineering Michigan State University East Lansing Michigan USA; ^3^ Sibley School of Mechanical and Aerospace Engineering Cornell University Ithaca New York USA; ^4^ U.S. Army Corps of Engineers Engineer Research and Development Center Construction Engineering Research Laboratory Champaign Illinois USA

**Keywords:** convection, hydrogel membrane, hydraulic pressure, porous medium, polymer

## Abstract

Molecular transport through polymer networks, including hydrogels and biological matrices, underpins many applications ranging from water filtration and gas separation to drug delivery and cell culture. Conventional strategies for regulating transport in polymer networks primarily focus on tuning molecular diffusion through network mesh size and polymer chemistry, whereas convection is often considered negligible because nanoscale‐mesh networks typically exhibit low fluid permeability. Although hydraulic pressure is a well‐established driving force for convection in porous media, extending pressure‐driven convection to non‐porous polymer networks has remained fundamentally challenging because they can undergo substantial deformation or fracture under pressure gradients. Here, we demonstrate that hydraulic pressure applied across mechanically tough and grid‐supported hydrogels enables robust and tunable solute transport while maintaining structural integrity. The characteristic transport time can be experimentally modulated by up to 65‐fold, consistent with a coupled diffusion–convection model. Beyond tuning transport kinetics, applied pressure enhances size‐ and charge‐dependent transport selectivity by up to 5.4‐fold compared to pressure‐free conditions. As a proof of concept, we demonstrate pressure‐programmed antimicrobial delivery that dynamically controls doxorubicin transport while blocking bacterial penetration. These findings identify pressure‐regulated convection as an underexplored mechanism for controlling transport in polymer networks.

## Graphical abstract

Hydraulic pressure is introduced as a programmable parameter for regulating molecular transport in tough hydrogel membranes. Reversing the pressure direction accelerates or slows transport, tuning characteristic transport times over a 65‐fold range while achieving molecular separation and controlled delivery.

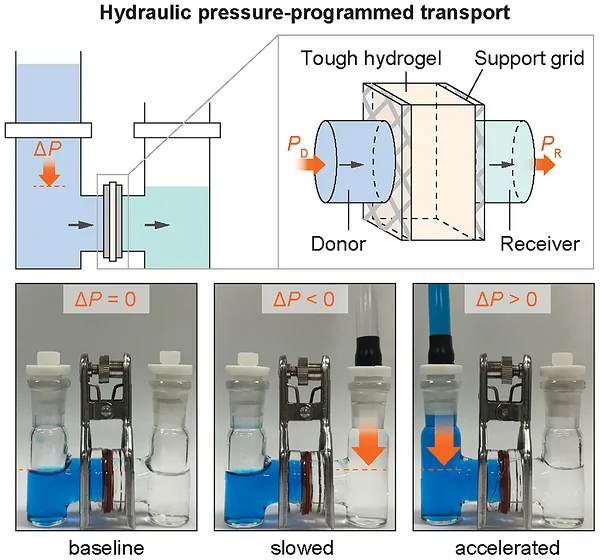

## 1 Introduction

Mass transport through soft materials plays a critical role in a wide range of biological processes, including nutrient delivery and waste removal in tissues, gas exchange in lungs, and drug distribution through blood circulation [1, 2, 3]. In these natural processes, molecular transport is generally governed by the coupled effects of diffusion and convection [4, 5, 6]. While diffusion enables slow, dispersive molecular transport driven by concentration gradients, convection allows rapid and directional transport through bulk fluid flow driven by pressure gradients [7, 8, 9]. Similarly, many engineered systems exploit the coupling between diffusion and convection, such as porous filtration membranes, chromatographic separation, and lab‐on‐a‐chip microfluidic devices [10, 11, 12]. In these cases, mechanically robust membranes containing interconnected micro‐scale pores allow efficient pressure‐regulated transport: under negligible pressure gradients, molecular transport is primarily governed by passive diffusion following Fick's law [13, 14], which is inherently slow and limited in tunability for a given membrane material; when hydraulic pressure is applied across the porous membrane, pressure‐driven convective flow substantially enhances transport rate and molecular separation efficiency following Darcy's law [15, 16, 17, 18]. The relatively high permeability of these microporous materials (pore size: 0.1–10 µm; permeability: 10^−12^–10^−8^ m^2^) enables strong convective transport under broadly tunable pressure gradients ranging from Pa to MPa [10, 19, 20, 21].

Hydrogels have been widely used in drug delivery, cell culture, and biosensing where they serve as matrix materials that host a wide range of active components (e.g., water, therapeutic molecules, proteins, and living cells) [22, 23, 24, 25]. The ability to regulate the transport of these active components is critical for controlling the dynamics of therapeutic delivery, cellular viability, and biosignal exchange within hydrogel systems [26]. Unlike microporous membranes with permeabilities of 10^−12^ to 10^−8^ m^2^, non‐porous hydrogels consisting of crosslinked polymer networks with nanoscale mesh sizes (1–5 nm) exhibit intrinsically low hydraulic permeability (*k* = 10^−18^ to 10^−15^ m^2^) [27, 28, 29]. As a result, transport in hydrogels is generally considered diffusion‐dominated, with pressure‐driven convection remaining negligible in comparison [24]. This contrast can be illustrated by considering a 1‐mm‐thick non‐porous hydrogel under a modest hydraulic pressure of 100 Pa: while the characteristic diffusion timescale is on the order of hours, the characteristic convection timescale extends to days, confirming that convective transport is effectively negligible in pressure‐free or low‐pressure hydrogel systems [29, 30, 31].

Despite the diffusion‐dominated transport under pressure‐free or low‐pressure conditions, sufficiently large pressure gradients can drive fluid migration through hydrogels, either generated internally by deformation or imposed externally across the material (Table S1) [31, 32, 33, 34]. During poroelastic processes, swelling, compression, or indentation induces transient internal pressure gradients that redistribute solvent within the polymer network [31, 33, 35]. Nevertheless, because these pressure gradients are intrinsically coupled to network deformation and solvent migration [32], they are difficult to independently prescribe and sustain as a controllable parameter. Alternatively, externally imposed pressure differences have been used to drive fluid flow through soft biological matrices such as collagen and fibrin gels [36, 37]. These studies, however, typically focus on interstitial flow under small, physiologically relevant pressure differences (0.01–1 kPa), rather than exploiting pressure as a broadly tunable parameter for regulating molecular transport [38, 39, 40]. Moreover, such soft matrices generally have limited mechanical robustness and are susceptible to irreversible deformation, crack propagation, and eventual structural failure under sustained hydraulic loading [41]. Thus, the systematic modulation of molecular transport through non‐porous hydrogels using independently controlled and broadly tunable hydraulic pressure remains unexplored. Recent advances in tough hydrogels provide a pathway to overcome these limitations [42, 43]. Through energy‐dissipation mechanisms such as sacrificial bond rupture in double‐network hydrogels and stress redistribution through topological entanglement, tough hydrogels exhibit substantially enhanced mechanical stability and fracture resistance under persistent loading [42, 44, 45]. These mechanically robust hydrogel systems therefore enable the systematic study of pressure‐driven convective transport in non‐porous polymer networks.

**FIGURE 1:**
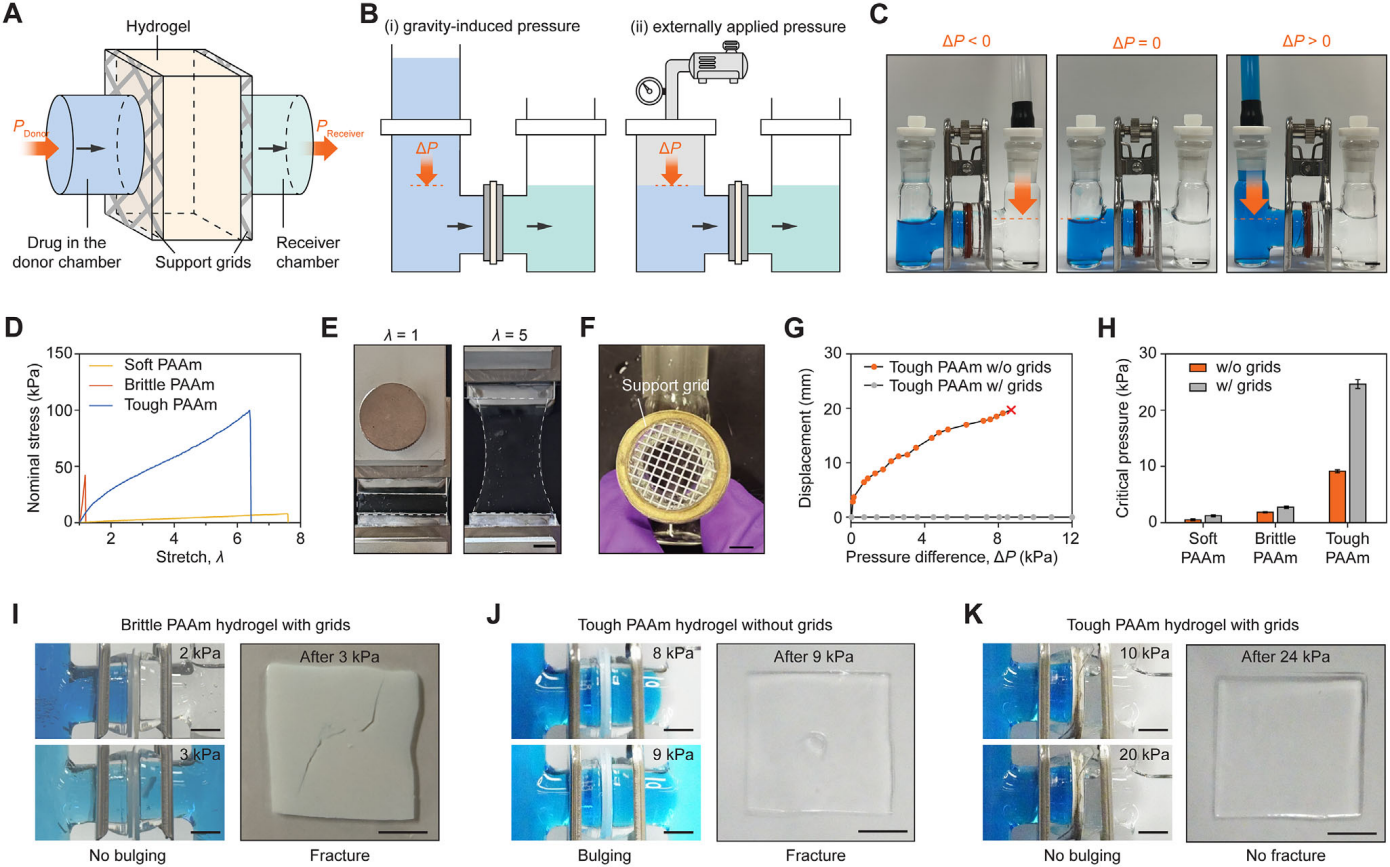
Pressure‐regulated molecular transport across mechanically tough and grid‐supported hydrogels. A) Schematic of hydraulic pressure–regulated molecular transport across a hydrogel membrane separating a donor chamber (*P*_Donor_) and a receiver chamber (*P*_Receiver_). Two support grids are attached to the hydrogel membrane to maintain structural stability under applied pressure differences. B) Two approaches for generating pressure differences (Δ*P*) across the hydrogel, including (i) hydrostatic pressure induced by liquid height differences and (ii) externally applied pressure using a compressed‐air system. C) Experimental demonstration of pressure regulation using liquid height differences to generate negative (Δ*P* < 0), zero (Δ*P* = 0), and positive (Δ*P* > 0) pressure differences across the hydrogel membrane in an H‐cell configuration. Scale bars: 10 mm. D) Stress–stretch curves of soft, brittle, and tough PAAm hydrogels. E) Images of a tough PAAm hydrogel under uniaxial tension at different stretches (*λ* = 1 and 5). Scale bars: 10 mm. F) Image of a chamber‐attached support grid that maintains the hydrogel's structural integrity. Scale bar: 10 mm. G) Out‐of‐plane displacement of tough PAAm hydrogels as a function of pressure difference (Δ*P*) in an H‐cell chamber with and without support grids. H) Critical pressure required to induce mechanical fracture and fluid leakage for soft, brittle, and tough PAAm hydrogels with and without support grids. I) Brittle PAAm hydrogels with support grids exhibit no deformation but fracture under a very small pressure difference (Δ*P* = 3 kPa). J) Tough PAAm hydrogels without support grids exhibit bulging and fracture under intermediate pressure differences (Δ*P* = 8–9 kPa). K) Tough PAAm hydrogels with support grids maintain structural integrity without bulging or fracture even at large pressure difference (Δ*P* = 24 kPa). Scale bars in (I,J,K): 10 mm.

Here, we identified hydraulic pressure as a key parameter for regulating molecular transport across tough hydrogels. We first developed an H‐cell platform in which a tough hydrogel membrane was mounted between donor and receiver chambers and mechanically stabilized using two microporous support grids (Figure 1A). Hydraulic pressure differences were applied either through liquid height differences or external pressurization using a compressed‐air system (Figure 1B). Using this experimental platform, we showed that the characteristic transport time could be modulated by up to 65‐fold as the applied pressure differences varied from −18 to 24 kPa, consistent with our coupled diffusion–convection model. We further demonstrated that this pressure‐regulated transport mechanism is applicable to different matrix chemistries (including neutral and charged hydrogels) and different transported species (ranging from small molecules of 1.5 nm to proteins of 7 nm). In addition to regulating the transport rate of a single species, applied pressure enhanced molecular separation by increasing size‐ and charge‐dependent transport selectivity between two different species by up to 5.4‐fold compared with pressure‐free conditions. As a proof‐of‐concept application, pressure‐programmed transport enabled selective antimicrobial delivery across the hydrogel membrane while preventing bacterial penetration. Taken together, these results established pressure‐regulated convection as an effective mechanism for controlling molecular transport and separation in non‐porous polymer networks.

## 2 Results

### 2.1 Tough, Grid‐Supported Hydrogels Under Hydraulic Pressure

To investigate the effects of hydraulic pressure on molecular transport, we constructed an H‐cell platform consisting of a donor chamber, a receiver chamber, and a non‐porous hydrogel membrane clamped between the two chambers (Figure 1A). Brilliant Blue FCF (Blue) was selected as a model solute for real‐time visualization and quantification. Initially, the donor chamber contained a higher concentration of solute (0.03 mg mL^−1^), while the receiver chamber contained none, driving solute diffusion across the hydrogel membrane from donor to receiver over time. Hydraulic pressure differences were generated by two complementary methods. In the first, a liquid‐height difference (*h*) between the two chambers produced a gravity‐induced pressure difference following Δ*P* = *ρgh*, where *ρ* is the liquid density and *g* is the gravitational acceleration. This configuration achieved pressure differences ranging from −18 to 24 kPa (Figure 1B, left). In the second, an externally regulated compressed‐air system enabled dynamic, time‐dependent control of the applied hydraulic pressure difference over a broader range (Figure 1B, right). Throughout this study, the pressure difference across the hydrogel membrane was defined as Δ*P = P*_Donor_ *−P*_Receiver_, where Δ*P* < 0, Δ*P* = 0, and Δ*P* > 0 correspond to lower donor pressure, no pressure difference, and higher donor pressure, respectively (Figure 1C). For the passive, gravity‐induced configuration, water transport across the hydrogel membrane gradually alters the liquid levels and, consequently, the pressure difference over extended periods (Figure S1). Therefore, the reported pressure values for these experiments refer to the initial pressure differences. In contrast, the externally regulated system actively maintains the prescribed pressure difference over time.

We next searched for hydrogel systems capable of sustaining hydraulic loading without significant deformation or fracture. Conventional polyacrylamide (PAAm) hydrogels synthesized with a low monomer concentration (14–20 wt% acrylamide) and variable crosslinker concentrations (0.002–5.3 wt% *N*,*N'*‐methylenebisacrylamide) are either soft (i.e., low modulus) or brittle (i.e., high modulus but low maximum strain) (Table S2). Therefore, these hydrogels exhibit limited tensile strength (7–35 kPa) and fracture toughness (10–160 J m^−2^) (Figure 1D; Figure S2). Under hydraulic loading, soft hydrogels readily deform, whereas brittle hydrogels fracture even under relatively small pressure differences. To overcome these limitations, we adopted a recently developed tough PAAm hydrogel formulation in which dense chain entanglements were introduced using an unusually high monomer concentration of 50 wt% [42, 44]. The resulting tough hydrogel exhibited substantially enhanced tensile strength (97 kPa) and fracture toughness (830 J m^−2^) due to efficient energy dissipation within the entangled polymer network (Figure 1D,E; Figure S2). To further suppress the hydraulic pressure‐induced deformation, two plastic support grids were placed on both sides of the hydrogel membrane to redistribute stress and minimize membrane bulging (Figure 1F). Although the grid struts reduced the open area by approximately 20%, the same grids were consistently used for all experiments to ensure reliable comparison of transport kinetics. By quantifying the displacement at the membrane center under different pressure differences, we found that the support grids effectively prevented deformation and increased the critical pressure that the hydrogel membranes could sustain from 9 to 24 kPa (Figure 1G,H).

We further compared the pressure response of different hydrogel conditions. Brittle PAAm hydrogels with support grids exhibited minimal deformation but experienced catastrophic membrane failure and uncontrolled fluid leakage under a small pressure difference of Δ*P* = 3 kPa (Figure 1I). Tough PAAm hydrogels without support grids exhibited substantial bulging deformation at Δ*P* = 8 kPa and fractured through the formation of a circular crack at Δ*P* = 9 kPa (Figure 1J). In contrast, tough PAAm hydrogels with support grids maintained structural integrity without noticeable bulging, fracture, or fluid leakage even under large pressure differences up to Δ*P* = 24 kPa (Figure 1K). These results demonstrate that both hydrogel toughness and mechanical support grids are necessary to achieve stable pressure‐regulated transport across non‐porous hydrogel membranes.

### 2.2 Pressure‐Controlled Transport Across Hydrogel Membranes

**FIGURE 2:**
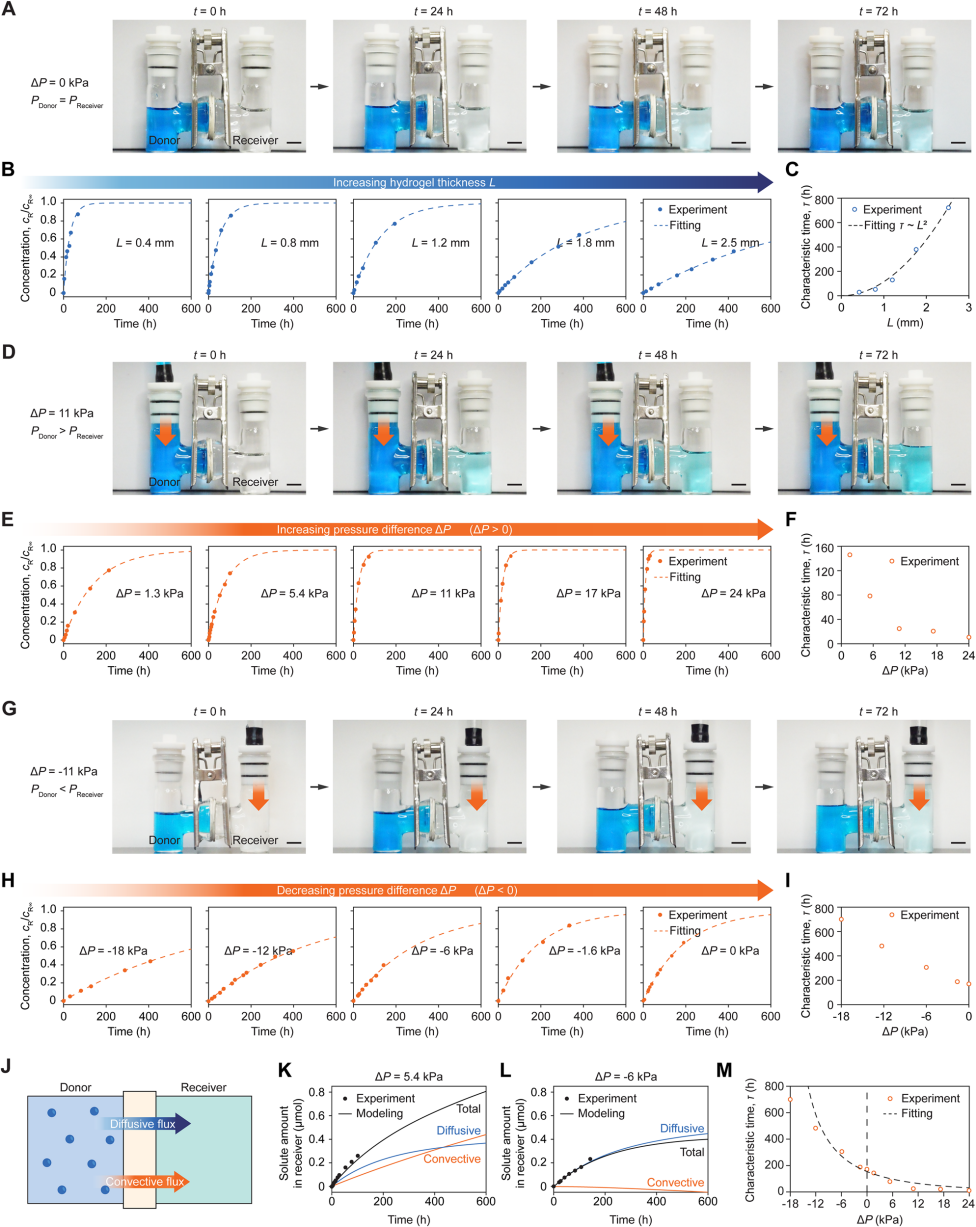
Effect of hydraulic pressure on diffusion–convection coupled transport across hydrogel membranes. A) Time‐series images showing molecular transport from the donor chamber to the receiver chamber across a hydrogel membrane without an applied hydraulic pressure difference (Δ*P* = 0 kPa, *P*_Donor_ *= P*_Receiver_). Hydrogel membrane thickness: 0.5 mm. B) Normalized receiver concentration (*c*_R_/*c*_R,∞_) as a function of time for hydrogel membranes with different thicknesses (0.4–2.5 mm) under zero applied pressure difference. C) Extracted characteristic time *τ* as a function of hydrogel membrane thickness *L*. Fitting confirms that *τ* scales approximately with *L* ^2^. D) Time‐series images showing molecular transport from the donor chamber to the receiver chamber across a hydrogel membrane under a positive hydraulic pressure difference (Δ*P* = 11 kPa, *P*_Donor_ *> P*_Receiver_). Hydrogel membrane thickness: 0.5 mm. E) Normalized receiver concentration (*c*_R_/*c*_R,∞_) as a function of time for hydrogel membranes under different positive hydraulic pressure differences (from 1.3 to 24 kPa). F) Extracted characteristic time *τ* as a function of applied pressure difference Δ*P*. G) Time‐series images showing molecular transport from the donor chamber to the receiver chamber across a hydrogel membrane under a negative hydraulic pressure difference (Δ*P* = −11 kPa, *P*_Donor_ < *P*_Receiver_). Hydrogel membrane thickness: 0.5 mm. H) Normalized receiver concentration (*c*_R_/*c*_R,∞_) as a function of time for hydrogel membranes under different negative hydraulic pressure differences (from −18 to 0 kPa). I) Extracted characteristic time *τ* as a function of applied pressure difference Δ*P*. J) Transport of solute molecules across the hydrogel membrane is a coupled process of diffusion and convection. K) Measured and calculated total quantity of solute transported to the receiver chamber as a function of time under a positive hydraulic pressure difference (Δ*P* = 5.4 kPa), together with the corresponding diffusive and convective contributions. L) Measured and calculated total quantity of solute transported to the receiver chamber as a function of time under a negative hydraulic pressure difference (Δ*P* = −6 kPa), together with the corresponding diffusive and convective contributions. M) Characteristic time *τ* as a function of applied pressure difference Δ*P*. Symbols represent experimental measurements and the dashed line represents fitting using a diffusion–convection coupled model. Scale bars in (A,D,G): 10 mm.

We systematically investigated molecular transport across mechanically tough, grid‐supported hydrogel membranes using the H‐cell platform. The concentration of model solute Blue in the receiver chamber was monitored by UV–Vis spectroscopy at 630 nm (Figure S3). Initially, the receiver chamber contained pure water, whereas the donor chamber contained Blue at a higher concentration. Over time, the solute concentration in the receiver chamber (*c*_R_) gradually increased until reaching the equilibrium concentration (*c*_R,∞_) equal to that in the donor chamber (*c*_D,∞_). In the absence of an applied pressure difference (*P*_Donor_ *= P*_Receiver_), molecular transport occurred through passive diffusion across the hydrogel membrane (Figure 2A). Increasing the hydrogel membrane thickness substantially slowed the transport process (Figure 2B). The time‐dependent concentration evolution in the receiver chamber was fitted using

$$\;\frac{c_{R}}{c_{R,\infty }}=1-e^{-t/\tau }$$

This first‐order exponential function provides a simple measure of the characteristic transport timescale *τ* across the hydrogel membrane [7, 8]. The physical basis of this functional form is further supported by the analytical solution of the diffusion–convection model (Note S1). The extracted transport time increased from approximately 30 h for a 0.4‐mm‐thick membrane to 720 h for a 2.5‐mm‐thick membrane. The extracted characteristic time *τ* scaled approximately with *L* ^2^, consistent with diffusion‐dominated transport across the pressure‐free hydrogel membrane (Figure 2C).

To apply positive hydraulic pressure differences, we created a higher water column on the donor side such that the donor chamber pressure exceeded that of the receiver chamber (*P*_Donor_ > *P*_Receiver_ or Δ*P* > 0). Under positive pressure differences, molecular transport was substantially accelerated compared with pressure‐free conditions (Figure 2A,D). Increasing the pressure difference from 0 kPa to 24 kPa progressively reduced the characteristic transport time from 189 to 11 h, indicating enhanced convective transport across the hydrogel membrane (Figure 2E,F). In contrast, creating a higher water column on the receiver side generated negative pressure differences (*P*_Donor_ *< P*_Receiver_ or Δ*P* < 0). This significantly slowed molecular transport, increasing the characteristic transport time from 189 h at Δ*P* = 0 to 700 h at Δ*P* = –18 kPa (Figure 2A,G–I). These results demonstrate that hydraulic pressure can either enhance or suppress molecular transport depending on the direction of the applied pressure gradient. Notably, the pressure dependence of the characteristic transport time did not strictly follow the inverse scaling predicted from Darcy‐driven convection alone (*τ* ∼ 1/ Δ*P*) (Figure 2F,I). Instead, the observed transport behavior reflects the coupled contributions of pressure‐driven convection and passive diffusion across the hydrogel membrane.

To provide a mechanistic and quantitative explanation for the observed pressure‐dependent transport, we considered the total molecular flux as the sum of diffusive and convective contributions: *J*_total_ = *J*_diff_ + *J*_conv_ (Figure 2J). Diffusive flux transports solute molecules (here Blue) from the high‐concentration donor chamber to the low‐concentration receiver chamber following Fick's law, *J*_diff_ = –*D*∇*C*, where *D* is the solute diffusivity and ∇*C* is the solute concentration gradient [13, 14]. In contrast, pressure‐driven convection follows Darcy's law, *J*_conv_ = –*k*∇*PC/µ*, where *k* is the hydraulic permeability, *µ* is the fluid viscosity, ∇*P* is the pressure gradient, and *C* is the local solute concentration [15, 16, 17, 18]. The hydraulic permeability (*k* = 1.27 × 10^−18^ m^2^) and diffusivity (*D* = 1.32 × 10^−11^ m^2^ s^−1^) were independently determined from water permeation and diffusion‐only experiments, respectively (Figure S4), and were used as fixed inputs to the coupled diffusion–convection model. The model quantitatively predicts both solvent and solute transport. The pressure‐driven solvent transfer follows:

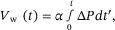

where *α* is a constant determined by the system geometry and transport properties (Note S1). At short times, Δ*P* remains approximately constant, and the transferred solvent volume therefore changes linearly with time (Figure S1). The corresponding solute concentration in the receiver chamber follows:

$$c_{R}\;\left(t\right)=c_{R,\infty }\;\left(1-e^{-\beta t}\right),$$

where *β* depends on the system geometry and the combined diffusive and convective transport contributions (Note S1). The calculated solvent‐volume and solute‐concentration profiles quantitatively agreed with the experimental results across different pressure conditions (Figure 2K,L; Figure S1). This model shows that, at Δ*P* = 0 kPa, transport is governed solely by diffusion. Under a positive pressure difference, convection acts in the same direction as diffusion and accelerates donor‐to‐receiver transport (Figure 2K). Conversely, under a negative pressure difference, convection opposes diffusion and delays donor‐to‐receiver transport (Figure 2L). From the analytical solution, the characteristic solute transport time is given by

$$\tau =\frac{V_{R}V_{D}L}{A\left[D\left(V_{D}+V_{R}\right)+\frac{k\Delta P}{\mu }\min\left(V_{D},V_{R}\right)\right]}$$

where *V*_D_ and *V*_R_ are the liquid volumes in the donor and receiver chambers used to establish the prescribed pressure difference, respectively, *A* is the cross‐sectional transport area, and *L* is the hydrogel membrane thickness. The model quantitatively captured the characteristic transport times across the entire pressure range (Figure 2M; Note S1). Across the pressure range from −18 to 24 kPa, the characteristic transport time was modulated from approximately 700 to 11 h, demonstrating hydraulic pressure as an effective parameter for regulating molecular transport kinetics across hydrogel membranes.

To contextualize this tunability, we compared the transport‐time tuning ratio, defined as *τ*_max_/*τ*_min_, with those of reported stimuli‐regulated transport systems based on temperature, external fields, and mechanical deformation (Figure S5). While most reported systems exhibit tuning ratios below 5‐fold [46, 47, 48, 49, 50, 51, 52, 53, 54, 55], and our recent mechanically regulated system achieved approximately 25‐fold tunability [56], the pressure‐regulated diffusion–convection coupling demonstrated here achieves a transport‐time tuning ratio of up to 65‐fold, representing an unusually broad dynamic range relative to previously reported strategies. In this model, permeability (*k*) and diffusivity (*D*) were assumed to remain constant across the applied pressure range, because the model solute is small and pressure‐induced hydrogel deformation was limited by the support grids. For larger solutes or larger local deformations, changes in mesh structure may affect both transport parameters and should be considered (Figure S6). Sensitivity analysis further confirmed that the predicted pressure‐dependent transport behavior was robust to variations in permeability (Figure S7).

### 2.3 Pressure‐Amplified Selective Transport

**FIGURE 3:**
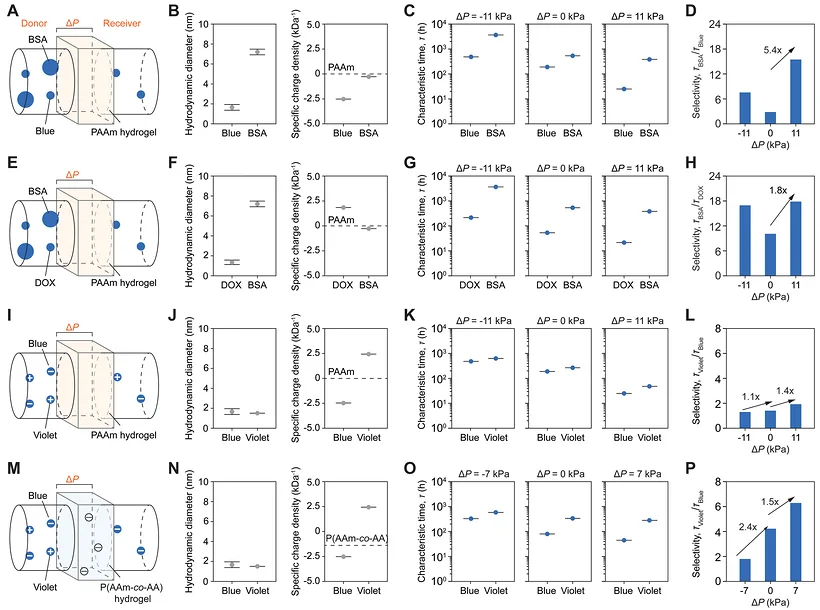
Pressure‐enhanced size‐ and charge‐selective molecular transport across hydrogel membranes. A) Schematic of selective transport between Blue and BSA across a PAAm hydrogel membrane under an applied hydraulic pressure difference. B) Hydrodynamic diameter and specific charge density of Blue and BSA relative to the PAAm hydrogel. C) Extracted characteristic time *τ* for Blue and BSA under applied pressure differences (Δ*P* = −11, 0, 11 kPa). D) Transport selectivity between BSA and Blue (*τ*_BSA_/*τ*_Blue_) as a function of pressure difference Δ*P*. A positive pressure difference enhances size‐dependent transport selectivity by 5.4‐fold compared to pressure‐free conditions (Δ*P* = 0 kPa). E) Schematic of selective transport between DOX and BSA across a PAAm hydrogel membrane under an applied hydraulic pressure difference. F) Hydrodynamic diameter and specific charge density of DOX and BSA relative to the PAAm hydrogel. G) Extracted characteristic time *τ* for DOX and BSA under applied pressure differences (Δ*P* = −11, 0, 11 kPa). H) Transport selectivity between BSA and DOX (*τ*_BSA_/*τ*_DOX_) as a function of pressure difference Δ*P*. A positive pressure difference enhances size‐dependent transport selectivity by 1.8‐fold compared to pressure‐free conditions (Δ*P* = 0 kPa). I) Schematic of selective transport between Blue and Violet across a PAAm hydrogel membrane under an applied hydraulic pressure difference. J) Hydrodynamic diameter and specific charge density of Blue and Violet relative to the PAAm hydrogel. K) Extracted characteristic time *τ* for Blue and Violet under applied pressure differences (Δ*P* = −11, 0, 11 kPa). L) Transport selectivity between Violet and Blue (*τ*_Violet_/*τ*_Blue_) as a function of pressure difference Δ*P*. Compared to pressure‐free conditions (Δ*P* = 0 kPa), a positive pressure difference (Δ*P* = 11 kPa) enhances charge‐dependent transport selectivity by 1.4‐fold, while a negative pressure difference (Δ*P* = −11 kPa) reduces the charge‐dependent transport selectivity by 1.1‐fold. M) Schematic of selective transport between Blue and Violet across a P(AAm‐*co*‐AA) hydrogel membrane under an applied hydraulic pressure difference. N) Hydrodynamic diameter and specific charge density of Blue and Violet relative to the P(AAm‐*co*‐AA) hydrogel. O) Extracted characteristic time *τ* for Blue and Violet under applied pressure differences (Δ*P* = −7, 0, 7 kPa). P) Transport selectivity between Violet and Blue (*τ*_Violet_/*τ*_Blue_) as a function of pressure difference Δ*P*. Compared to pressure‐free conditions (Δ*P* = 0 kPa), a positive pressure difference (Δ*P* = 7 kPa) enhances charge‐dependent transport selectivity by 1.5‐fold, while a negative pressure difference (Δ*P* = −7 kPa) reduces the charge‐dependent transport selectivity by 2.4‐fold.

Selective transport based on solute size or charge is widely utilized in separation engineering. To study pressure‐regulated selectivity, we first focused on size‐dependent transport using Blue, doxorubicin (DOX), and bovine serum albumin (BSA) in PAAm hydrogels (Figure 3A–H; Table S3) [57, 58, 59, 60, 61, 62, 63]. Blue and DOX are small molecules with hydrodynamic diameters of 1.68 and 1.35 nm and specific charge densities of −2.52 and +1.84 kDa^−1^, respectively. BSA is a protein macromolecule with a hydrodynamic diameter of 7.2 nm. BSA and the PAAm hydrogel matrix are nearly neutral under the experimental conditions (Figure 3B,F; Table S3). Therefore, the Blue/BSA and DOX/BSA pairs were used to investigate size‐selective transport under applied hydraulic pressure. BSA exhibited substantially longer characteristic transport times than Blue and DOX (*τ*_BSA_ >> *τ*_Blue_ and *τ*_BSA_ >> *τ*_DOX_) because the larger protein experienced greater steric resistance during transport through the PAAm network (Figure 3C,G; Figure S8). Consistent with earlier observations, increasing Δ*P* from −11 to 0 and then to 11 kPa progressively reduced the characteristic transport times of all solutes (Figure 3C,G). To quantify selective transport, we defined selectivity as the ratio of characteristic transport times between two solutes under the same pressure condition. Under pressure‐free conditions (Δ*P* = 0 kPa), BSA exhibited substantially slower transport than Blue and DOX, resulting in *τ*_BSA_/*τ*_Blue_ = 2.8 and *τ*_BSA_/*τ*_DOX_ = 10.1 due to steric hindrance during diffusion‐dominated transport. Applying either negative or positive pressure differences further enhanced selective transport. For example, *τ*_BSA_/*τ*_Blue_ increased by 2.7‐fold at −11 kPa and 5.4‐fold at 11 kPa relative to the zero‐pressure condition (Figure 3D). Similarly, pressure application increased *τ*_BSA_/*τ*_DOX_ by approximately 1.7–1.8‐fold (Figure 3H).

We further investigated charge‐selective transport using Blue and crystal violet (Violet), two small‐molecule dyes with comparable hydrodynamic diameters (1.68 and 1.51 nm) but opposite specific charge densities of –2.52 and +2.45 kDa^−1^, respectively (Figure 3I,J; Table S3) [57, 60]. Using electrically neutral PAAm hydrogels, we observed only weak selectivity between the two dyes (*τ*_Violet_/*τ*_Blue_ = 1.4) under pressure‐free conditions (Δ*P* = 0 kPa). Sweeping the applied pressure from –11 to 11 kPa accelerated the transport of both molecules but maintained relatively low selectivity (*τ*_Violet_/*τ*_Blue_ < 2) because the neutral hydrogel network interacted similarly with both charged solutes (Figure 3K,L; Figure S9). To further evaluate electrostatic effects on selective transport, we synthesized negatively charged P(AAm‐*co*‐AA) hydrogels containing 10 mol% acrylic acid (AA) and 90 mol% acrylamide, corresponding to a specific charge density of –1.39 kDa^−1^. Because incorporation of charged acrylic acid reduced the mechanical strength of the hydrogel, the applied pressure range was limited to ±7 kPa. Under pressure‐free conditions (Δ*P* = 0 kPa), Violet exhibited substantially slower transport than Blue, resulting in a selectivity of τ_Violet_/τ_Blue_ = 4.2 (Figure 3O,P; Figure S9). This behavior is likely caused by electrostatic attraction between positively charged Violet and the negatively charged hydrogel matrix [30]. Interestingly, negative pressure reduced the selectivity to ∼1.8, whereas positive pressure increased the selectivity to ∼6.3 (Figure 3P). The overall selectivity increased by 3.5‐fold when the pressure was switched from –7 to 7 kPa.

To understand pressure‐regulated selectivity, particularly its enhancement under positive pressure, we performed a theoretical analysis based on the diffusion–convection model (Note S2 and Figure S10). At zero pressure, selectivity is determined by the diffusivity contrast between two solutes (*D*_1_/*D*_2_). Under a positive pressure difference (Δ*P* > 0), molecule‐specific convective hindrance introduces an additional source of discrimination, and the selectivity progressively shifts toward the effective permeability contrast (*k*_1_/*k*_2_) as convection becomes increasingly dominant. Therefore, positive pressure enhances selectivity when the permeability contrast exceeds the diffusivity contrast (*k*_1_/*k*_2_ > *D*_1_/*D*_2_). This condition can arise from different molecular mechanisms. For size selectivity, larger solutes experience stronger steric obstruction and higher energy barriers during transport through the polymer network. Pressure‐induced changes in mesh structure may further amplify this difference because solutes approaching or exceeding the mesh size are substantially more sensitive to network deformation than smaller solutes (Figure S6) [56]. For charge selectivity, electrostatic attraction between oppositely charged solutes and the polymer network increases solute retention and reduces effective permeability relative to that of similarly charged solutes. Together, these size‐ and charge‐dependent differences in effective permeability explain why pressure‐driven convection can amplify molecular selectivity beyond that achieved by diffusion alone.

### 2.4 Pressure‐Programmed Drug Delivery

**FIGURE 4:**
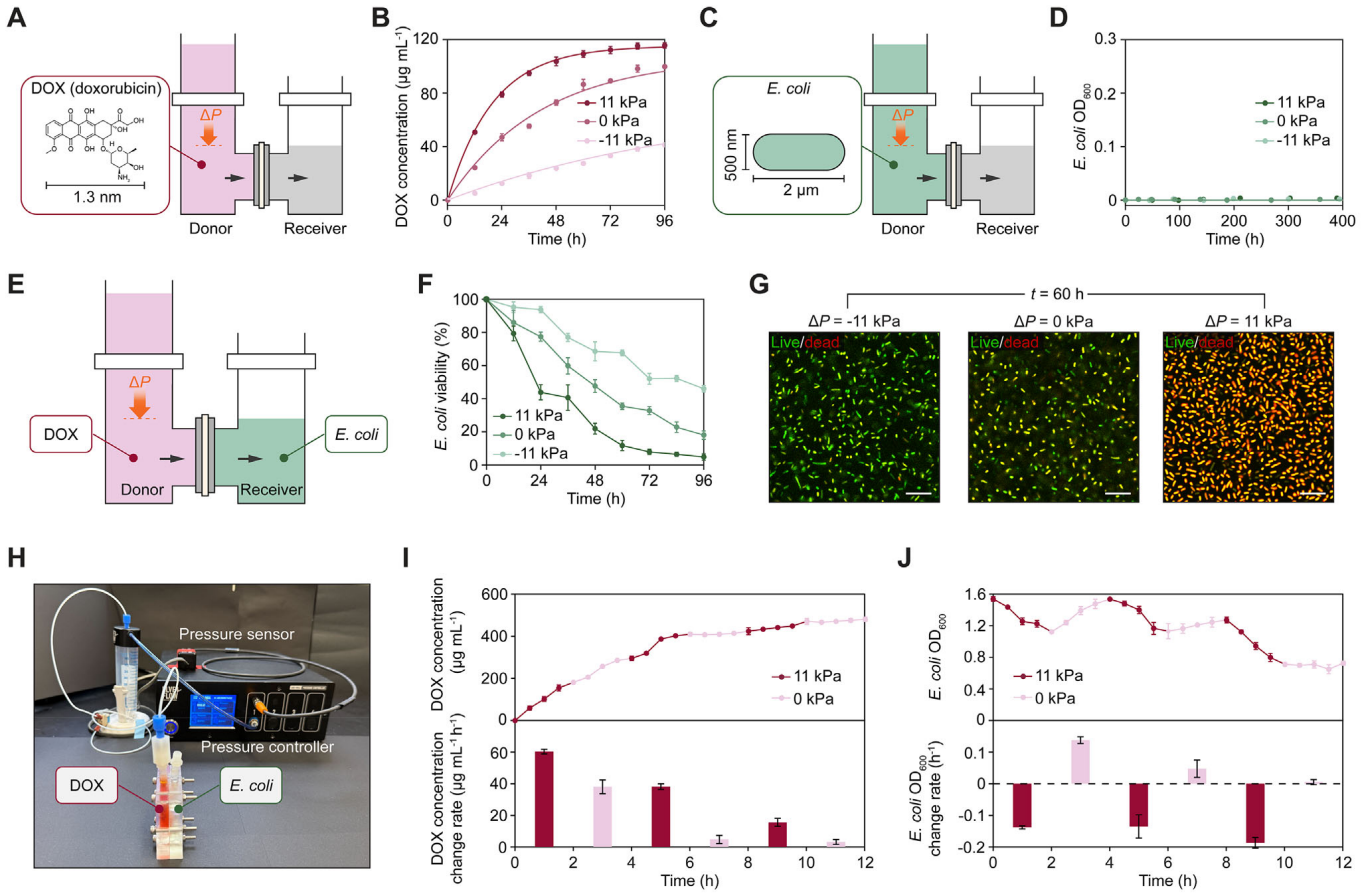
Pressure‐programmed antimicrobial delivery across hydrogel membranes. A) Schematic of pressure–regulated DOX transport across a hydrogel membrane under applied hydraulic pressure differences. B) DOX concentration in the receiver chamber as a function of time under different hydraulic pressure differences (Δ*P* = −11, 0, 11 kPa). C) Schematic of pressure–regulated *E. coli* transport across a hydrogel membrane under applied hydraulic pressure differences. D) *E. coli* optical density (OD_600_) in the receiver chamber as a function of time under different hydraulic pressure differences (Δ*P* = −11, 0, 11 kPa). E) Schematic of pressure–programmed antimicrobial delivery, where DOX is transported from the donor to the receiver while *E. coli* is confined to the receiver chamber. F) *E. coli* viability in the receiver chamber as a function of time under different hydraulic pressure differences (Δ*P* = −11, 0, 11 kPa). G) Representative live/dead fluorescence images of *E. coli* after 60 h under different applied hydraulic pressure differences (Δ*P* = −11, 0, 11 kPa). Scale bars: 100 µm. H) Image of the pressure‐programmed antimicrobial delivery platform integrated with a pressure‐control system. I) DOX concentration and corresponding concentration change rate in the receiver chamber over time under alternating hydraulic pressure differences (Δ*P* = 11 kPa for 2 h and 0 kPa for 2 h). J) *E. coli* OD_600_ and corresponding *E. coli* OD_600_ change rate in the receiver chamber over time under alternating hydraulic pressure differences (Δ*P* = 11 kPa for 2 h and 0 kPa for 2 h). Data are presented as mean ± standard deviation (*N* = 3 technical replicates in B and *N* = 3 biological replicates in D, F, I and J).

To further demonstrate the practical relevance of pressure‐regulated transport, we investigated pressure‐programmed antimicrobial delivery of DOX to *Escherichia coli* (*E. coli*) cultures. DOX, a small‐molecule antimicrobial agent with a hydrodynamic diameter of 1.35 nm [58, 59], exhibited strongly pressure‐dependent transport behavior, with the characteristic transport time varying by approximately 10‐fold across the pressure range from −11 to 11 kPa (Figure 4A,B; Figure S8). Under a positive pressure difference, the DOX concentration in the receiver chamber reached an effective bacteria‐inhibiting concentration of ∼50 µg mL^−1^ within 12 h, corresponding to a >99% reduction in *E. coli* density (Figure 4B; Figure S11). In contrast, a negative pressure difference substantially slowed DOX transport, and even after 48 h, the transported concentration remained below 30 µg mL^−1^, which was insufficient to fully suppress *E. coli* survival and growth (Figure 4B; Figure S11). Unlike DOX, *E. coli* are living bacteria with a characteristic size of 0.5–2 µm [64], making them too large to cross the non‐porous hydrogel membrane (mesh size: 1–5 nm) under all tested pressure conditions (Figure 4C,D) [27, 28, 29]. In the absence of DOX, more than 95% of *E. coli* cells remained viable in the receiver chamber (Figure S11). To evaluate pressure‐programmed antimicrobial delivery, *E. coli* cells were cultured in the receiver chamber while DOX was initially loaded in the donor chamber separated by the hydrogel membrane (Figure 4E). At Δ*P* = 11 kPa, efficient DOX transport resulted in strong antibacterial activity, with >88% of *E. coli* cells dead after 60 h, as confirmed by live/dead staining (Figure 4F,G) [65, 66]. In contrast, at Δ*P* = –11 kPa, limited DOX transport led to substantially higher bacterial survival (>67% viability, Figure 4F,G).

These results suggest that hydraulic pressure can program the temporal profiles of antimicrobial delivery and bacterial inhibition, which is critical for treating infections under dynamically evolving physiological conditions [67]. To further demonstrate dynamic pressure‐controlled delivery, we developed a 3D‐printed H‐cell device in which the donor chamber containing DOX was connected to a pressure‐controlled air compressor, while the receiver chamber containing *E. coli* remained under atmospheric pressure (Figure 4H; Figure S12). The pressure in the donor chamber was alternated between 0 and 11 kPa every two hours to dynamically regulate molecular transport across the hydrogel membrane. Consistent with previous observations, the DOX concentration in the receiver chamber gradually increased over time toward equilibrium (Figure 4I). During each 0–11 kPa pressure cycle, the DOX transport rate was consistently higher at 11 kPa than at 0 kPa, showing temporally programmable molecular transport under dynamic pressure control (Figure 4I). Correspondingly, *E. coli* growth also strongly depended on the time‐evolving antimicrobial transport. During the 0 kPa intervals, bacterial growth continued as indicated by increasing optical density (OD), whereas during the 11 kPa intervals, bacterial growth was effectively suppressed and the OD decreased over time (Figure 4J).

## 3 Discussion

In this study, we demonstrated hydraulic pressure as an effective external parameter for regulating molecular transport across hydrogel membranes. Although molecular transport in hydrogels is generally considered diffusion‐dominated because of their nanoscale mesh size and ultralow hydraulic permeability, our results showed that pressure‐driven convection can substantially alter transport kinetics when sustained pressure gradients are applied. Mechanically tough and grid‐supported hydrogels enabled pressure differences ranging from −18 to 24 kPa to be sustained for extended periods (>400 h) without substantial deformation, fracture, or fluid leakage. Through a combination of experimental characterization and coupled diffusion–convection modeling, we revealed that the hydraulic pressure can modulate the characteristic transport time by up to 65‐fold and amplify size‐ and charge‐dependent transport selectivity by up to 5.4‐fold. The mechanism is applicable across several different hydrogel chemistries, membrane thicknesses, and transported species, establishing pressure‐regulated convection as an underexplored strategy for controlling molecular transport in polymer networks.

These findings provide fundamental insights into how externally applied hydraulic pressure regulates molecular transport within non‐porous polymer networks, advancing our understanding of diffusion–convection coupling in soft materials and guiding the design of next‐generation hydrogel systems with mechanically programmable transport and separation capabilities. Potential applications include intelligent wound dressings, localized drug delivery, adaptive tissue engineering scaffolds, high‐selectivity biosensing substrates, and soft microfluidic systems requiring temporally controlled and spatially selective molecular exchange under dynamically varying mechanical environments. Beyond these biomedical applications, the reversible and pressure‐programmable transport behavior also opens new opportunities for electrochemical interfaces and chemical energy conversion systems, where dynamically regulated molecular and ionic transport is critical for controlling reaction kinetics and system efficiency.

Several limitations and future directions remain to be explored. First, the present study primarily focused on the role of steady and low‐frequency hydraulic pressure in regulating convective transport. Future work should investigate pressure‐regulated transport under cyclic and physiologically relevant loading conditions, including pulsatile pressure profiles commonly found in biological tissues and vascular systems. Second, extending this approach to a broader range of hydrogel systems, such as anisotropic polymer networks and biologically active matrices, may provide new insights into the coupling among network architecture, mechanical deformation, and molecular transport. Such extensions will require material‐specific optimization to balance mechanical robustness with transport responsiveness. Third, the current model assumes homogeneous material properties and does not explicitly resolve spatially heterogeneous mechanical deformation or molecular‐scale interactions within the polymer network. Future computational work integrating finite‐element and molecular dynamics simulations could further elucidate how local network deformation and molecular interactions regulate transport parameters and govern nonequilibrium diffusion–convection coupling.

## Author Contributions

Y.C., L.Z., S.H., and X.L. designed research; Y.C., S.A., A.Z., Z.H., S.H., and X.L. performed research; Y.C., S.A., A.Z., Z.H., J.Z., S.H., and X.L. analyzed data; and Y.C. and X.L. wrote the paper.

## Conflicts of Interest

The authors declare no conflict of interest.

## Supporting information




**Supporting File**: advs77159‐sup‐0001‐SuppMat.pdf.

## Data Availability

The data that support the findings of this study are available from the corresponding author upon reasonable request.
