# Supplementary material for: Hydraulic Pressure‐Programmed Molecular Transport in Tough Hydrogels
Source: Adv Sci (Weinh). 2026 Aug 20;13(60):e77159. doi: 10.1002/advs.77159 (PMC13491361; doi:10.1002/advs.77159)
Supplement: Supplementary file 1 [file ADVS-13-e77159-s001.pdf]

Supporting Information for

Hydraulic Pressure-Programmed Molecular Transport in Tough Hydrogels

Yijie Cheng[1], Shahd Alnasser[2], Akhiri Zannat[1], Zhenda He[1], Jiayi Zeng[3], Lenan Zhang[3], Sungmin Hong[4], and Xinyue Liu[1,*]

[1]Department of Chemical Engineering and Materials Science, Michigan State University, East Lansing, MI 48824, United States

[2]Department of Mechanical Engineering, Michigan State University, East Lansing, MI 48824, United States

[3]Sibley School of Mechanical and Aerospace Engineering, Cornell University, Ithaca, NY 14853

[4]U.S. Army Corps of Engineers, Engineer Research and Development Center, Construction Engineering Research Laboratory, Champaign, IL, USA

*Corresponding authors. Emails: xyliu@msu.edu

This PDF file includes:

Materials and Methods

Notes S1 to S2

Figures S1 to S12

Tables S1 to S3

References

Materials and Methods

Synthesis of PAAm hydrogel membranes

Three types of polyacrylamide (PAAm) hydrogels were synthesized using the formulations listed in **Table S2**. To prepare the tough PAAm hydrogel, an acrylamide (AAm) precursor solution was prepared by dissolving 5 g of AAm monomer in 5 mL of deionized water. Subsequently, 800 μL of *N,N′*-methylenebisacrylamide (MBAA) aqueous solution (0.23 wt.%) as the crosslinker, 40 μL of ammonium persulfate (APS) aqueous solution (0.1 M) as the thermal initiator, and 20 μL of *N,N,N′,N′*-tetramethylethylenediamine (TEMED) as the polymerization accelerator were added to the precursor solution. The mixture was purged with nitrogen for 2 min to remove dissolved oxygen and then injected into a custom-designed silicone spacer sandwiched between two glass slides. The hydrogel membrane thickness was controlled by the thickness of the silicone spacer. The mixture was thermally crosslinked in an oven at 75 °C for 1 h, which turned the solution to a solid hydrogel. After crosslinking, the hydrogels were carefully removed from the glass slides and immersed in deionized water until fully swollen before use. Similarly, the soft and brittle PAAm hydrogels were prepared following a similar procedure using the corresponding formulations listed in **Table S2**.

Synthesis of P(AAm-*co*-AA) hydrogel membranes

The negatively charged poly(acrylamide-*co*-acrylic acid) (P(AAm-*co*-AA)) hydrogel was synthesized. First, the precursor solution was prepared by dissolving 0.5 g of acrylic acid (AA) and 4.5 g of AAm in 5 mL of deionized water. Subsequently, 800 μL of MBAA aqueous solution (0.23 wt.%) as the crosslinker, 40 μL of APS aqueous solution (0.1 M) as the thermal initiator, and 20 μL of TEMED as the polymerization accelerator were added into the precursor solution. The mixture was purged with nitrogen for 2 min to remove dissolved oxygen and then injected into a custom-designed silicone spacer sandwiched between two glass slides. The hydrogel membrane thickness was controlled by the thickness of the silicone spacer. The mixture was thermally crosslinked in an oven at 75 °C for 1 h, which turns the solution to a solid hydrogel. After crosslinking, the hydrogels were carefully removed from the glass slides and immersed in deionized water until fully swollen before use.

Mechanical characterization

Different PAAm hydrogel formulations were synthesized, fully swollen in water, and cut into rectangular pure-shear specimens.(*1-3*) Both ends of each specimen were secured using customized acrylic holders and mounted onto a mechanical testing machine (CMT6000, Sansi) with a testing width of 40 mm, a thickness of 1.0 mm, and a gauge length of 10 mm. Tensile tests were conducted at a constant loading rate of 1 mm s^{-1} for paired unnotched and notched specimens until failure. For the notched specimens, an initial 10-mm-

long crack was introduced perpendicular to the loading direction. The critical stretch, λ_c, was determined from the notched specimens at the onset of steady-state crack propagation. The stress–stretch curves of the corresponding unnotched specimens were then used to obtain strength (the maximum stress) and fracture toughness Γ following the equation (*1-3*)

$$\Gamma = H \int_1^{\lambda_c} \sigma d\lambda$$

where H is the initial gauge length of the specimen, σ is the nominal stress, and λ is the stretch ratio.

Glass H-cell platform

An open-access, dual-compartment glass membrane cell (Corrtest), consisting of a donor chamber and a receiver chamber, was used as the H-cell platform for transport experiments. A fully swollen hydrogel membrane was sandwiched between two plastic support grids (9218T61, McMaster-Carr) and mounted between the donor and receiver chambers. The effective membrane area was 1.5 cm^2, and the membrane thickness was 0.5 mm unless otherwise specified. For gravity-driven hydrostatic pressure control, both chambers were open to atmospheric pressure. When the liquid levels in the two chambers were equal, no hydrostatic pressure difference was applied across the membrane (ΔP = 0 kPa). To generate a hydrostatic pressure difference, the liquid volume in one chamber was fixed at 10 mL, whereas the other chamber, connected to a vertical polyvinyl chloride (PVC) tube (A_{tube} = 0.8 cm^2, 51825K51, McMaster-Carr), was filled with a total liquid volume ranging from 10 to 205 mL, corresponding to a liquid-level difference of 0–248 cm between the two sides. This liquid-level difference (h) generated a hydrostatic pressure difference (ΔP) across the hydrogel membrane based on $\Delta P = \rho g h$, where ρ is the liquid density and g is the gravitational acceleration. Both chambers were covered with lids to minimize evaporation but were not hermetically sealed and remained exposed to atmospheric pressure. Using this setup, we characterized the out-of-plane displacement of the hydrogel membrane under different applied pressure differences. The critical pressure was defined as the applied pressure at which membrane fracture and liquid leakage occurred.

3D-printed plastic H-cell platform

For pneumatically controlled experiments, a custom-designed H-cell was fabricated using a 3D printer (Formlabs Form 3+). The H-cell consisted of donor and receiver chambers, each with a liquid capacity of 5 mL, with support grids monolithically printed as part of the chamber structures. A fully swollen hydrogel membrane was mounted between the two support grids, and the two halves of the H-cell were tightly assembled using screws. The donor chamber was connected to a pressure-control system consisting of a pressure sensor (MPS-V2-L-2, Elveflow), a pressure controller (OB1 MK4, Elveflow), and an air

compressor (1P10605, California Air Tools). The system provided programmable pressure control over a range of 0–800 kPa with a step resolution of 0.001 kPa. The receiver chamber was exposed to atmospheric pressure, thereby enabling direct control of the pressure difference across the hydrogel membrane.

Solute concentration measurements

Optical absorbance at a characteristic wavelength was used as a proxy for solute concentration in aqueous solution. A range of solutes, including Brilliant Blue FCF (Blue), doxorubicin (DOX), crystal violet (Violet), and bovine serum albumin (BSA), were dissolved in phosphate-buffered saline (PBS). *Escherichia coli* (*E. coli*) cells were dispersed in PBS. To establish the relationship between solute concentration and optical absorbance, UV–Vis absorbance spectra were measured using a NanoDrop One spectrophotometer (Thermo Scientific). The absorbance at a characteristic wavelength was used for solute concentration or bacterial cell density quantification: 630 nm for Blue, 480 nm for DOX, 590 nm for Violet, 280 nm for BSA, and 600 nm for *E. coli*. Calibration curves were established by preparing a series of solutions or suspensions with known solute concentrations or cell densities through serial dilution and measuring the corresponding absorbance at the characteristic wavelength. For each species, the characteristic absorbance was plotted as a function of known solute concentration or cell density and fitted using linear regression to obtain the calibration curve.

Transport kinetics characterization

For transport experiments, a solution or suspension containing the species of interest was introduced into the donor chamber at a prescribed initial concentration, $c_{\mathrm{D}}(0)$, including 0.03 mg mL^{-1} for Blue, 0.2 mg mL^{-1} for DOX, 0.01 mg mL^{-1} for Violet, 0.03 mg mL^{-1} for BSA, and an initial OD_{600} of 1.0 for *E. coli*. The receiver chamber was initially filled with pure PBS, corresponding to $c_{\mathrm{R}}(0) = 0$. Solute concentrations were monitored by sampling from the chamber containing a fixed liquid volume of 10 mL, which was the receiver chamber for $\Delta P > 0$ and the donor chamber for $\Delta P < 0$. At each sampling time point, a 10 μL aliquot was collected, and its absorbance was measured using a NanoDrop One spectrophotometer (Thermo Scientific). For each transport experiment, samples were collected at 6–12 time points over the entire experimental period, corresponding to a total sampling volume of 0.06–0.12 mL. This volume accounted for no more than 1.2% of the 10 mL initial liquid volume and was therefore considered negligible. For $\Delta P > 0$, the solute concentration in the receiver chamber $c_{\mathrm{R}}(t)$ was measured directly. For $\Delta P < 0$, the solute concentration in the donor chamber $c_{\mathrm{D}}(t)$ was measured, and the corresponding solute concentration in the receiver chamber $c_{\mathrm{R}}(t)$ was calculated from solute mass conservation as

$$c_{\mathrm{R}}(t) = \frac{V_{\mathrm{D}}(0)c_{\mathrm{D}}(0) - V_{\mathrm{D}}(t)c_{\mathrm{D}}(t)}{V_{\mathrm{R}}(t)}$$

where $V_{\mathrm{D}}(0)$ is the initial liquid volume in the donor chamber, $V_{\mathrm{D}}(t)$ and $V_{\mathrm{R}}(t)$ are the measured liquid volumes in the donor and receiver chambers at time t, respectively, and $c_{\mathrm{D}}(t)$ is the measured solute concentration in the donor chamber at time t. The solute concentration in the receiver chamber $c_{\mathrm{R}}(t)$ was normalized by the equilibrium solute concentration in the receiver chamber $c_{R,\infty}$. Assuming complete solute equilibration between the two chambers and conservation of the total solute mass, the equilibrium receiver concentration was calculated as

$$c_{R,\infty} = \frac{V_{\mathrm{D}}(0)c_{\mathrm{D}}(0)}{V_{\mathrm{D}}(0) + V_{\mathrm{R}}(0)}$$

The normalized concentration profiles were then fitted using a first-order exponential function, $c_{\mathrm{R}}/c_{\mathrm{R},\infty} = 1 - e^{-t/\tau}$ to extract the characteristic transport time τ across the hydrogel membrane.

Permeability measurement

The hydraulic permeability was independently quantified by measuring the volumetric water flux across the hydrogel membrane under a series of constant pressure differences (1− 20 kPa) applied using a compressed-air pressure regulator. According to Darcy's law, the volumetric water flux through the hydrogel membrane is given by (*4, 5*)

$$\frac{Q}{A} = \frac{k\Delta P}{\mu L},$$

where Q is the volumetric flow rate of water through the membrane, A is the effective membrane area, k is the hydraulic permeability of the hydrogel, μ is the dynamic viscosity of water, ΔP is the applied pressure difference across the membrane, and L is the membrane thickness. We measured the water flux under varied applied pressures and observed a linear relationship between the volumetric water flux Q/A and the pressure gradient $\Delta P/L$. According to Darcy's law, the slope of this relationship is k/μ. With the known water viscosity, we can independently calculate the hydraulic permeability.

Diffusivity measurement

Diffusivity was determined from the zero-pressure-difference (ΔP = 0 kPa) condition, where transport is governed solely by diffusion:

$$J = \frac{D}{L}[c_{\mathrm{D}}(t) - c_{\mathrm{R}}(t)],$$

where J is the molecular flux across the hydrogel membrane, D is the solute diffusivity in the hydrogel, L is the hydrogel membrane thickness, and $c_{\mathrm{D}}(t)$ and $c_{\mathrm{R}}(t)$ are the time-dependent solute concentrations in the donor and receiver chambers, respectively. Under this condition, the concentration evolution in the receiver chamber can be analytically described as

$$c_{\mathrm{R}}(t) = \frac{V_{\mathrm{D}} c_{\mathrm{D}}(0)}{V_{\mathrm{D}} + V_{\mathrm{R}}} \left(1 - e^{-\frac{DA(V_{\mathrm{D}} + V_{\mathrm{R}})}{V_{\mathrm{R}} V_{\mathrm{D}} L} t}\right)$$

where $c_{\mathrm{D}}(0)$ is the initial solute concentration in the donor chamber, V_{D} and V_{R} are the constant liquid volumes in the donor and receiver chambers, respectively, and A is the effective membrane transport area. Since V_{D}, V_{R}, L, and A are known experimentally, D can be calculated by fitting the analytical solution to the zero-pressure transport data, i.e., the normalized receiver concentration $c_{\mathrm{R}}/c_{\mathrm{R},\infty}$ as a function of time t.

E. coli cultivation

E. coli (ATCC 25922) was used as a representative bacterial strain for antimicrobial evaluation. A single colony from a freshly streaked Luria–Bertani (LB) agar plate was inoculated into LB broth and cultured overnight at 37 °C with shaking at 200 rpm. The bacterial culture was then collected and diluted to the cell density of OD_{600} = 1.0 using sterile PBS before use in subsequent experiments.

Antimicrobial test

The glass H-cell platform was used to evaluate pressure-regulated antimicrobial delivery, where the donor chamber was loaded with 10 mL of DOX solution in PBS (0.4 mg mL^{-1}), and the receiver chamber was loaded with 10 mL of an *E. coli* suspension in PBS (OD_{600} = 1.0). Three pressure conditions were established, including (1) a liquid-level difference of 112 cm established on the receiver side using the vertical tubing filled with the *E. coli* suspension for $\Delta P = -11$ kPa; (2) the liquid levels on the two sides were equal for $\Delta P = 0$ kPa; and (3) a liquid-level difference of 112 cm applied on the donor side using the vertical tubing filled with the DOX solution for $\Delta P = 11$ kPa. To quantify bacterial viability, a 10 µL aliquot of the bacterial suspension was collected from the receiver chamber every 12 h and diluted 10^4-fold with sterile PBS. Subsequently, 25 µL of the diluted suspension was spread onto sterile LB agar plates and incubated statically at 37 °C overnight. The agar plates were photographed using a digital camera, and the number of colony-forming units (CFU) was quantified using ImageJ software (National Institutes of Health, Bethesda, MD, USA). The *E. coli* viability was calculated as CFU(t)/CFU(0) × 100%, where CFU(t) and CFU(0) are the CFU of *E. coli* suspension in the receiver chamber at transport time t and at the beginning of the test, respectively. Bactericidal efficiency was further evaluated using live/dead fluorescence staining according to previous studies.(*6, 7*) At 60 h, 1 mL of bacterial suspension was collected from the receiver chamber and mixed with 15 µL of a staining solution containing acridine orange (AO, 1 mg mL^{-1}) and ethidium bromide (EB, 1 mg mL^{-1}). The samples were incubated in the dark at 4 °C for 15 min and then centrifuged at 5000 rpm for 5 min. The bacterial pellets were washed with sterile PBS to remove excess dye and resuspended in 100 µL of glycerol. A drop of the stained bacterial suspension was placed on a glass slide, covered with a coverslip, and sealed with nail polish. Fluorescence images were acquired using a

confocal laser scanning microscope (TCS SP8, Leica). Live bacteria stained with AO exhibited green fluorescence (λ_{ex} = 500 nm, λ_{em} = 525 nm), whereas dead bacteria stained with EB exhibited red fluorescence (λ_{ex} = 543 nm, λ_{em} = 625 nm).

Temporally programmable antimicrobial test

The 3D-printed H-cell platform connected to the pneumatic pressure-control system was used to evaluate temporally programmed drug release and its antimicrobial effect. The donor chamber was loaded with 5 mL of DOX solution in PBS (0.4 mg mL^{-1}), and the receiver chamber was loaded with 5 mL of an *E. coli* suspension in PBS (OD_{600} = 1.0). The pressure difference across the hydrogel membrane was programmed to alternate between ΔP = 11 kPa for 2 h and ΔP = 0 kPa for 2 h. Every 30 min, a 10 μL sample was collected from the donor and receiver chambers. The DOX concentration in the donor chamber was quantified by measuring its characteristic absorbance, while the bacterial density in the receiver chamber was monitored by OD_{600} to evaluate bacterial growth in response to pressure-programmed DOX release.

Supplementary Notes

Note S1. Diffusion–convection coupled modeling

I. Assumptions and variables

To describe the molecular transport across the hydrogel membrane in the H-cell platform, we assume that 1) the donor and receiver chambers are well mixed with spatially uniform solute concentration; 2) solute reaction, degradation, and adsorption are negligible, 3) the partition coefficient at the hydrogel–solution interfaces is unity, 4) transport within the hydrogel membrane is quasi-steady-state. The notation of variables used in the model is summarized in the following table.

Symbol	Definition	Unit
t	Time	s or h
τ	Characteristic solute transport time	s or h
x	Position across the membrane, measured from donor to receiver	m
L	Hydrogel membrane thickness	m
A	Effective membrane transport area	m^2
V_{D}	Liquid volume in the donor chamber	m^3
V_{R}	Liquid volume in the receiver chamber	m^3
c_{D}	Solute concentration in the donor chamber	mol m^{-3}
c_{R}	Solute concentration in the receiver chamber	mol m^{-3}
$c_{\mathrm{R},\infty}$	Equilibrium solute concentration in the receiver chamber	mol m^{-3}
D	Solute diffusivity in the hydrogel	m^2 s^{-1}
k	Hydraulic permeability of the hydrogel	m^2
μ	Dynamic viscosity of the solvent	Pa s
ρ	Mass density of the solvent	kg m^{-3}
g	Gravitational acceleration	m s^{-2}
ΔP	Hydraulic pressure difference (initial)	Pa
ΔP_{real}	Hydraulic pressure difference (real)	Pa
J_{diff}	Diffusive solute flux across the membrane	mol m^{-2} s^{-1}
J_{conv}	Convective solute flux across the membrane	mol m^{-2} s^{-1}
J	Total solute flux across the membrane	mol m^{-2} s^{-1}
M_{diff}	Solute increase due to diffusion in the receiver	mol
M_{conv}	Solute increase due to convection in the receiver	mol
M	Total solute increase in the receiver	mol
M_{D}	Solute quantity in the donor chamber	mol
M_{R}	Solute quantity in the receiver chamber	mol
Q	Volumetric solvent flow rate	m^3 s^{-1}
v	Volumetric solvent flow velocity	m s^{-1}
V_{w}	Cumulative solvent volume transported	m^3
h	Liquid-level difference generating the hydrostatic pressure	m
h_0	Initial liquid-level difference generating the initial hydrostatic pressure	m
α	Geometry factor relating solvent transfer to the liquid-level change	dimensionless
A_{tube}	Cross-sectional area of the pressure-generating tube	m^2

II. Solvent transport

In the hydrostatic-pressure glass H-cell configuration, solvent transport across the hydrogel membrane gradually reduces the liquid-level difference between the donor and receiver chambers, resulting in a time-dependent hydrostatic pressure difference. Here, we estimate the evolution of the pressure difference over time $\Delta P_{\mathrm{real}}(t)$ to evaluate whether the initial pressure difference ΔP can be used as a representative pressure condition during the transport experiments. Solvent transport across the hydrogel membrane is described by Darcy's law.(*4, 5*) The volumetric solvent flow rate is given by

$$\frac{Q(t)}{A} = \frac{k\Delta P_{\mathrm{real}}(t)}{\mu L} \tag{1}$$

or equivalently,

$$Q(t) = A\frac{k\Delta P_{\mathrm{real}}(t)}{\mu L} \tag{2}$$

The cumulative solvent volume transported across the membrane is

$$V_{\mathrm{w}}(t) = \int_0^t Q(t')\,dt' \tag{3}$$

As solvent is transported through the membrane, the liquid-level difference gradually decreases according to

$$h(t) = h_0 - \alpha\frac{V_{\mathrm{w}}(t)}{A_{\mathrm{tube}}} \tag{4}$$

For the hydrostatic configuration, the pressure difference is generated by the liquid-level difference between the donor and receiver chambers,

$$\Delta P_{\mathrm{real}} = \rho g h(t) \tag{5}$$

The calculated pressure decay indicates that the hydrostatic pressure difference decreases by approximately 4% after 120 h under representative initial pressure differences of ΔP = –6 kPa or 5.4 kPa (**Figure S1**). This limited variation supports the use of the initial pressure difference ΔP as a representative pressure condition over the primary transport timescale. Under substantially longer durations or larger initial pressure differences, however, pressure decay becomes more significant. For example, for an initial pressure difference of ΔP = 24 kPa, the model predicts a pressure decrease of ~45% after 600 h (**Figure S1**).

III. Solute transport

To predict the time-dependent solute concentration evolution in the donor and receiver chambers under different applied pressure differences, solute transport across the hydrogel membrane was modeled as the combined effect of molecular diffusion and pressure-driven convection. The diffusive solute flux through the hydrogel membrane is described by Fick's law,(*8, 9*)

$$J_{\mathrm{diff}}(t) = \frac{D}{L}\left(c_{\mathrm{D}}(t) - c_{\mathrm{R}}(t)\right) \tag{6}$$

The convective solute flux is determined by the solvent flow through the membrane and therefore depends on the upstream solute concentration. Specifically,

$$J_{\mathrm{conv}} = \begin{cases} v(t)c_{\mathrm{D}}(t), & \Delta P \geq 0 \\ v(t)c_{\mathrm{R}}(t), & \Delta P < 0 \end{cases} \tag{7}$$

where the Darcy velocity is

$$v = \frac{k\Delta P_{\mathrm{real}}(t)}{\mu L} \tag{8}$$

Accordingly, the total solute flux across the membrane is

$$J = J_{\mathrm{diff}} + J_{\mathrm{conv}} \tag{9}$$

The donor and receiver chambers are coupled through mass conservation. The transient solute mass balances are

$$\frac{d(V_{\mathrm{D}}(t)c_{\mathrm{D}}(t))}{dt} = -AJ(t) \tag{10}$$

in the donor chamber and

$$\frac{d(V_{\mathrm{R}}(t)c_{\mathrm{R}}(t))}{dt} = AJ(t) \tag{11}$$

in the receiver chamber. The liquid volumes in the two chambers $V_{\mathrm{R}}(t)$ and $V_{\mathrm{D}}(t)$ were treated as time dependent in the numerical model to account for solvent transport across the membrane. However, the estimated volume change $V_{\mathrm{w}}(t)$ and pressure difference $\Delta P_{\mathrm{real}}(t)$ change during the experimental timescale was negligible (**Figure S1**). We use ΔP instead of $\Delta P_{\mathrm{real}}(t)$ to represent the near-constant pressure difference. Therefore, for deriving the analytical solution, the liquid volumes in the two chambers were approximated as constants, giving

$$V_{\mathrm{D}}\frac{dc_{\mathrm{D}}(t)}{dt} = -AJ(t) \tag{12}$$

and

$$V_{\mathrm{R}}\frac{dc_{\mathrm{R}}(t)}{dt} = AJ(t) \tag{13}$$

Substituting the total solute flux (Eqs. (6), (7), and (8)) into the receiver mass balance (Eq. (13)) gives

For $\Delta P \geq 0$,

$$V_{\mathrm{R}}\frac{dc_{\mathrm{R}}(t)}{dt} = A\left[\frac{D}{L}(c_{\mathrm{D}}(t) - c_{\mathrm{R}}(t)) + \frac{k\Delta P}{\mu L}c_{\mathrm{D}}(t)\right] \tag{14}$$

For $\Delta P < 0$,

$$V_{\mathrm{R}}\frac{dc_{\mathrm{R}}(t)}{dt} = A\left[\frac{D}{L}\left(c_{\mathrm{D}}(t) - c_{\mathrm{R}}(t)\right) - \frac{k\Delta P}{\mu L}c_{\mathrm{R}}(t)\right] \tag{15}$$

To eliminate $c_{\mathrm{D}}(t)$ in the governing equation, we consider that the receiver chamber contains no solute initially,

$$c_{\mathrm{R}}(0) = 0 \tag{16}$$

Because the total solute mass is conserved in the two chambers,

$$V_{\mathrm{D}} c_{\mathrm{D}}(t) + V_{\mathrm{R}} c_{\mathrm{R}}(t) = V_{\mathrm{D}} c_{\mathrm{D}}(0) \tag{17}$$

which gives

$$c_{\mathrm{D}}(t) = \frac{V_{\mathrm{D}} c_{\mathrm{D}}(0) \ - V_{\mathrm{R}} c_{\mathrm{R}}(t)}{V_{\mathrm{D}}} \tag{18}$$

Substituting the expression for $c_{\mathrm{D}}(t)$ (Eq. (18)) into the governing equations (Eqs. (14) and (15)) yields

For $\Delta P \geq 0$,

$$\frac{dc_{\mathrm{R}}(t)}{dt} = \frac{A}{V_{\mathrm{R}} V_{\mathrm{D}} L} \left[\left(D + \frac{k\Delta P}{\mu} \right) V_{\mathrm{D}} c_{\mathrm{D}}(0) - \left(D(V_{\mathrm{D}} + V_{\mathrm{R}}) + \frac{k\Delta P}{\mu} V_{\mathrm{R}} \right) c_{\mathrm{R}}(t) \right] \tag{19}$$

For $\Delta P < 0$,

$$\frac{dc_{\mathrm{R}}(t)}{dt} = \frac{A}{V_{\mathrm{R}} V_{\mathrm{D}} L} \left[D V_{\mathrm{D}} c_{\mathrm{D}}(0) - \left(D(V_{\mathrm{D}} + V_{\mathrm{R}}) + \frac{k\Delta P}{\mu} V_{\mathrm{D}} \right) c_{\mathrm{R}}(t) \right] \tag{20}$$

Both equations can be written in the general form

$$\frac{dc_{\mathrm{R}}(t)}{dt} = C_1 - C_2 c_{\mathrm{R}}(t) \tag{21}$$

where C_1 and C_2 are constants determined by the transport parameters and the applied pressure difference. With the initial condition ($c_{\mathrm{R}}(0) = 0$), the analytical solution for the differential equations (Eqs. (19) and (20)) is

$$c_{\mathrm{R}}(t) = \frac{C_1}{C_2}(1 - e^{-C_2 t}) \tag{22}$$

At equilibrium $t \to \infty$, we can obtain

$$c_{R,\infty} = \frac{C_1}{C_2} \tag{23}$$

and therefore the normalized concentration evolution in the receiver chamber can be expressed as

$$\frac{c_{\mathrm{R}}(t)}{c_{R,\infty}} = 1 - e^{-C_2 t} \tag{24}$$

where the characteristic transport time is defined as

$$\tau = \frac{1}{C_2} \tag{25}$$

Specifically, for $\Delta P \geq 0$,

$$\tau = \frac{V_{\mathrm{R}} V_{\mathrm{D}} L}{A \left(D(V_{\mathrm{D}} + V_{\mathrm{R}}) + \frac{k\Delta P}{\mu} V_{\mathrm{R}} \right)} \tag{26}$$

and for $\Delta P < 0$,

$$\tau = \frac{V_\mathrm{R} V_\mathrm{D} L}{A\left(D(V_\mathrm{D} + V_\mathrm{R}) + \frac{k\Delta P}{\mu} V_\mathrm{D}\right)} \tag{27}$$

To quantify the respective contributions of diffusion and pressure-driven convection to solute transport, the cumulative amounts of solute transported by diffusion, convection, and their combined effect were calculated as

$$\begin{cases} M_\mathrm{diff}(t) = \int_0^t A J_\mathrm{diff}(t')dt' \\ M_\mathrm{conv}(t) = \int_0^t A J_\mathrm{conv}(t')dt' \\ M(t) = \int_0^t A J(t')dt' \end{cases} \tag{28}$$

Because the total solute flux is the sum of the diffusive and convective fluxes, that is,

$$J = J_\mathrm{diff} + J_\mathrm{conv} \tag{29}$$

the cumulative transported quantities satisfy

$$M = M_\mathrm{diff} + M_\mathrm{conv} \tag{30}$$

For $\Delta P > 0$, diffusion and convection act in the same direction, resulting in a positive convective contribution $M_\mathrm{conv}(t) > 0$. For $\Delta P < 0$, convection opposes diffusion, resulting in a negative convective contribution $M_\mathrm{conv}(t) < 0$. At $\Delta P = 0$, the convective contribution vanishes $M_\mathrm{conv}(t) = 0$ and the total transported amount is determined solely by diffusion.

The solute quantity in the donor chamber is

$$M_\mathrm{D}(t) = V_\mathrm{D}(0)c_\mathrm{D}(0) - M(t) \tag{31}$$

The solute quantity in the receiver chamber is

$$M_\mathrm{R}(t) = M(t) \tag{32}$$

IV. MATLAB simulation

The analytical solution derived above assumes constant liquid volumes in receiver and donor chambers (V_R and V_D) and a constant pressure difference (ΔP). This approximation accurately captures the experimental transport kinetics and enables extraction of the characteristic transport time. However, pressure-driven solvent transport gradually redistributes solvent between the donor and receiver chambers, causing both the chamber volumes and the hydrostatic pressure difference to evolve with time, particularly under large initial pressure differences and over long observation periods. Therefore, V_R, V_D, and ΔP must be treated as time-dependent variables.

Because the two chambers are connected through vertical tubes of constant cross-sectional area, the transferred solvent volume is directly related to the liquid-height difference. The chamber volumes and hydrostatic pressure satisfy

$$V_{\mathrm{D}}(t) - V_{\mathrm{R}}(t) = \alpha A_{\mathrm{tube}} h(t) \quad (33)$$

where α is a geometry factor accounting for the relationship between the transferred solvent volume and the resulting liquid-height difference. The corresponding hydrostatic pressure difference is

$$\Delta P_{\mathrm{real}}(t) = \rho g h(t) \quad (34)$$

Consequently, solvent transport continuously reduces the liquid-height difference and the associated hydrostatic pressure, producing a time-dependent decay in the driving force. To account for this pressure decay, the coupled diffusion–convection governing equations (Eqs. (10) and (11)) were solved numerically in MATLAB to obtain the temporal evolution of liquid volumes, pressure difference, solute concentrations, and cumulative diffusive and convective transport.

Note S2. Mechanistic analysis of pressure-regulated solute selectivity

To provide a more quantitative explanation for the observed pressure-enhanced selectivity between two different solutes, we derived an analytical expression based on the diffusion–convection transport model and further performed a simplified numerical analysis using experimentally measured transport parameters. In brief, pressure enhances selectivity when the permeability contrast between two solutes exceeds their diffusivity contrast ($k_1/k_2 > D_1/D_2$), such that increasing pressure shifts transport from diffusion-dominated behavior toward convection-dominated behavior that more strongly discriminates between the two solutes. The solute-dependent permeability contrast can arise from size-dependent steric hindrance or charge-dependent electrostatic interactions with the hydrogel network, thereby providing a common framework for understanding both size- and charge-selective transport. For an arbitrary solute *i*, the transport flux can be expressed as

$$J_i = \frac{D_i}{L}\left(c_{\mathrm{D},i}(\mathrm{t}) - c_{\mathrm{R},i}(t)\right) + \frac{k_i \Delta P}{\mu L} c_{\mathrm{D},i}(t) \tag{35}$$

where D_i is the solute diffusivity and k_i is the solute-specific effective permeability.

I. Theoretical analysis of pressure-enhanced size-selectivity

We first consider two solutes with markedly different molecular sizes. The diffusivity needs to account for the pressure-dependent mesh size change, especially for large-sized solute, and the permeability accounts for pressure-driven convection and convective hindrance within the hydrogel network. Using the same derivation as described above, the characteristic transport time can be approximated as

$$\tau_i = \frac{V_\mathrm{R} V_\mathrm{D} L}{A\left[D_i + \frac{k_i \Delta P}{\mu}\frac{\min(V_\mathrm{D}, V_\mathrm{R})}{V_\mathrm{D} + V_\mathrm{R}}\right]} \tag{36}$$

Therefore, the size selectivity between a large and a small solute is

$$\frac{\tau_\mathrm{large}}{\tau_\mathrm{small}} = \frac{D_\mathrm{small} + \frac{k_\mathrm{small}\Delta P}{\mu}\frac{\min(V_\mathrm{D}, V_\mathrm{R})}{V_\mathrm{D} + V_\mathrm{R}}}{D_\mathrm{large} + \frac{k_\mathrm{large}\Delta P}{\mu}\frac{\min(V_\mathrm{D}, V_\mathrm{R})}{V_\mathrm{D} + V_\mathrm{R}}} \tag{37}$$

where D_large and D_small denote the diffusivities of the large-sized and small-sized solutes, respectively, whereas k_large and k_small are their effective permeabilities. Because the two solutes differ significantly in molecular size, the larger solute exhibits both a lower diffusivity ($D_\mathrm{large} < D_\mathrm{small}$) and a lower effective permeability ($k_\mathrm{large} < k_\mathrm{small}$) than the smaller solute.

The term $\min(V_\mathrm{D}, V_\mathrm{R})$ in Eq. (37) depends on the direction of the applied pressure difference. When $\Delta P \geq 0$, the receiver chamber is maintained at a small, fixed liquid volume, whereas the donor chamber has a substantially larger liquid volume to generate the gravity-induced hydrostatic pressure. Therefore, the characteristic minimum liquid volume is $\min(V_\mathrm{D}, V_\mathrm{R}) = V_\mathrm{R}$. Conversely, when $\Delta P < 0$, the donor chamber

remains at a small, fixed liquid volume and the characteristic minimum liquid volume $\min(V_\mathrm{D}, V_\mathrm{R}) = V_\mathrm{D}$. Based on Eq. (37), three cases can be readily identified below:

Case i: Zero pressure difference

When $\Delta P = 0$, transport is entirely diffusion-controlled,

$$\frac{\tau_\mathrm{large}}{\tau_\mathrm{small}} = \frac{D_\mathrm{small}}{D_\mathrm{large}} \tag{38}$$

Therefore, the size selectivity is determined solely by the difference in diffusivity.

Case ii: Positive pressure difference

When $\Delta P > 0$, convection acts in the same direction as diffusion, increasing the transport rates of both solutes. In the convection-dominated limit,

$$\frac{k_i \Delta P}{\mu} \frac{V_\mathrm{R}}{V_\mathrm{D} + V_\mathrm{R}} \gg D_i \tag{39}$$

the selectivity approaches

$$\frac{\tau_\mathrm{large}}{\tau_\mathrm{small}} \to \frac{k_\mathrm{small}}{k_\mathrm{large}} \tag{40}$$

Thus, pressure enhances size selectivity when

$$\frac{k_\mathrm{small}}{k_\mathrm{large}} > \frac{D_\mathrm{small}}{D_\mathrm{large}} \tag{41}$$

because the pressure-driven convective contribution discriminates more strongly between small and large solutes than diffusion alone.

Case iii: Negative pressure difference

When $\Delta P < 0$, pressure-driven convection opposes diffusion. The effective transport driving force becomes

$$D_i - \frac{k_i |\Delta P|}{\mu} \frac{V_\mathrm{D}}{V_\mathrm{D} + V_\mathrm{R}} \tag{42}$$

Unlike the positive-pressure limit, negative pressure does not yield a universal asymptotic selectivity because the relative importance of diffusion and opposing convection depends on both D_i and k_i. Consequently, the size selectivity under negative pressure is governed by the competition between diffusion and opposing convection.

Pressure-induced mesh deformation. The above analysis assumes that D_i and k_i remain constant. However, hydraulic loading may also induce local deformation of the hydrogel network, thereby changing the effective mesh structure. For the present system, Blue is substantially smaller than the hydrogel mesh size, whereas BSA has a hydrodynamic size comparable to or larger than the characteristic mesh size. According to obstruction and hopping diffusion models,(*10-12*) moderate mesh-size variations have only a small influence on the diffusivity of the small solute like Blue (**Figure S6A**), but produce a much stronger

effect on the large solute like BSA (**Figure S6B**). Therefore, D_{Blue} is assumed to be a pressure-independent constant, whereas D_{BSA} is dependent on the absolute value of ΔP and may decrease as pressure-induced deformation reduces the effective mesh structure.

II. Numerical simulation of pressure-enhanced size-selectivity

For the Blue–BSA pair in a PAAm hydrogel, we used the transport parameters under zero-pressure conditions including D_{small} = 1.32 × 10^{-11} m^2 s^{-1} and k_{small} = 1.27 × 10^{-18} m^2 for Blue, and D_{large} = 2.87 × 10^{-12} m^2 s^{-1} and k_{large} = 2.54 × 10^{-20} m^2 for BSA. To illustrate the proposed mechanism, a minimal model was adopted in which D_{small}, k_{small}, and k_{large} were assumed to remain constant, whereas D_{large} was allowed to decrease with increasing $|\varDelta P|$, consistent with the theoretical prediction of deformation-dependent hopping diffusion (**Figure S6B**). The resulting model semi-quantitatively reproduced the experimentally observed amplification of size selectivity under both positive and negative pressure differences (**Figure S10A-C**). A similar analysis for the DOX–BSA pair also qualitatively reproduced the experimentally observed pressure-enhanced selectivity (**Figure S10D–F**).

III. Theoretical analysis of pressure-enhanced charge-selectivity

We next consider two solutes with similar molecular sizes but opposite charges. Following the same derivation, the charge selectivity can be expressed as

$$\frac{\tau_+}{\tau_-} = \frac{D_- + \dfrac{k_- \varDelta P}{\mu} \dfrac{\min(V_{\mathrm{D}}, V_{\mathrm{R}})}{V_{\mathrm{D}} + V_{\mathrm{R}}}}{D_+ + \dfrac{k_+ \varDelta P}{\mu} \dfrac{\min(V_{\mathrm{D}}, V_{\mathrm{R}})}{V_{\mathrm{D}} + V_{\mathrm{R}}}} \tag{43}$$

where D_+ and D_- denote the diffusivities of the positively and negatively charged solutes, respectively, whereas k_+ and k_- are their effective permeabilities. Because the two solutes have similar molecular sizes, their diffusivities are expected to be comparable ($D_+ \approx D_-$). However, electrostatic interactions between the charged solutes and the negatively charged hydrogel network lead to substantially different effective permeabilities. The positively charged solute experiences stronger attractive electrostatic interactions with the negatively charged hydrogel network, resulting in a much stronger retention of positive solutes than the negative solutes ($k_- \gg k_+$). Consequently, hydraulic pressure primarily amplifies the convective contribution rather than the diffusive contribution, thereby enhancing charge selectivity. Specifically, three cases are identified.

Case i: Zero pressure difference

When $\varDelta P = 0$, transport is governed solely by diffusion,

$$\frac{\tau_+}{\tau_-} = \frac{D_-}{D_+} \tag{44}$$

Because the two solutes have similar molecular sizes, D_+ and D_- are of the same order of magnitude. Therefore, the charge selectivity is primarily determined by the relatively small difference in diffusivity.

Case ii: Positive pressure difference

When $\Delta P > 0$, pressure-driven convection acts in the same direction as diffusion, thereby accelerating the transport of both solutes. Because the negatively charged solute experiences weaker electrostatic interactions with the negatively charged hydrogel network, its effective permeability is substantially higher than that of the positively charged solute ($k_- \gg k_+$). Consequently,

$$\frac{\tau_+}{\tau_-} = \frac{D_- + \frac{k_-\Delta P}{\mu}\frac{\min(V_D, V_R)}{V_D + V_R}}{D_+ + \frac{k_+\Delta P}{\mu}\frac{\min(V_D, V_R)}{V_D + V_R}} > \frac{D_-}{D_+} \tag{45}$$

Thus, positive pressure difference amplifies charge selectivity. This enhanced selectivity is because the two solutes possess similar diffusivities but markedly different effective permeabilities. As the pressure-driven convective contribution becomes more important, transport becomes increasingly dominated by the difference in effective permeability rather than diffusivity.

Case iii: Negative pressure difference

When $\Delta P < 0$, pressure-driven convection opposes diffusion. Substituting $\Delta P = -|\Delta P|$ gives

$$\frac{\tau_+}{\tau_-} = \frac{D_- - \frac{k_-|\Delta P|}{\mu}\frac{V_D}{V_D + V_R}}{D_+ - \frac{k_+|\Delta P|}{\mu}\frac{V_D}{V_D + V_R}} \tag{46}$$

Because $k_- \gg k_+$,

$$\frac{\tau_+}{\tau_-} < \frac{D_-}{D_+} \tag{47}$$

Thus, negative pressure suppresses charge selectivity because the opposing convective contribution reduces the transport rate of the negatively charged solute more strongly than that of the positively charged solute.

IV. Numerical simulation of pressure-enhanced charge-selectivity.

For the Blue–Violet pair in the negatively charged P(AAm-*co*-AA) hydrogel, we used the transport parameters under zero-pressure conditions, including D_- = 1.32 × 10^{-11} m^2 s^{-1} and k_-= 1.27 × 10^{-18} m^2 for Blue, and D_+ = 8.80 × 10^{-12} m^2 s^{-1} and k_+ = 0.64 × 10^{-18} m^2 for Violet. In the illustrative simulation, D_-, D_+, k_-, and k_+ were assumed to remain constant over the applied pressure range because both solutes are substantially smaller than the hydrogel mesh size and therefore are expected to exhibit negligible pressure-dependent changes in transport parameters (**Figure S6**). The resulting model semi-quantitatively reproduced the experimentally observed enhancement of charge selectivity under positive pressure difference only but suppressed selectively under negative pressure difference (**Figure S10J-L**). As a control, simulations in a neutral PAAm hydrogel predicted little pressure dependence of charge selectivity because the effective permeabilities of the two solutes are nearly identical in the absence of electrostatic interactions (**Figure S10G-I**).

Supplementary Figures

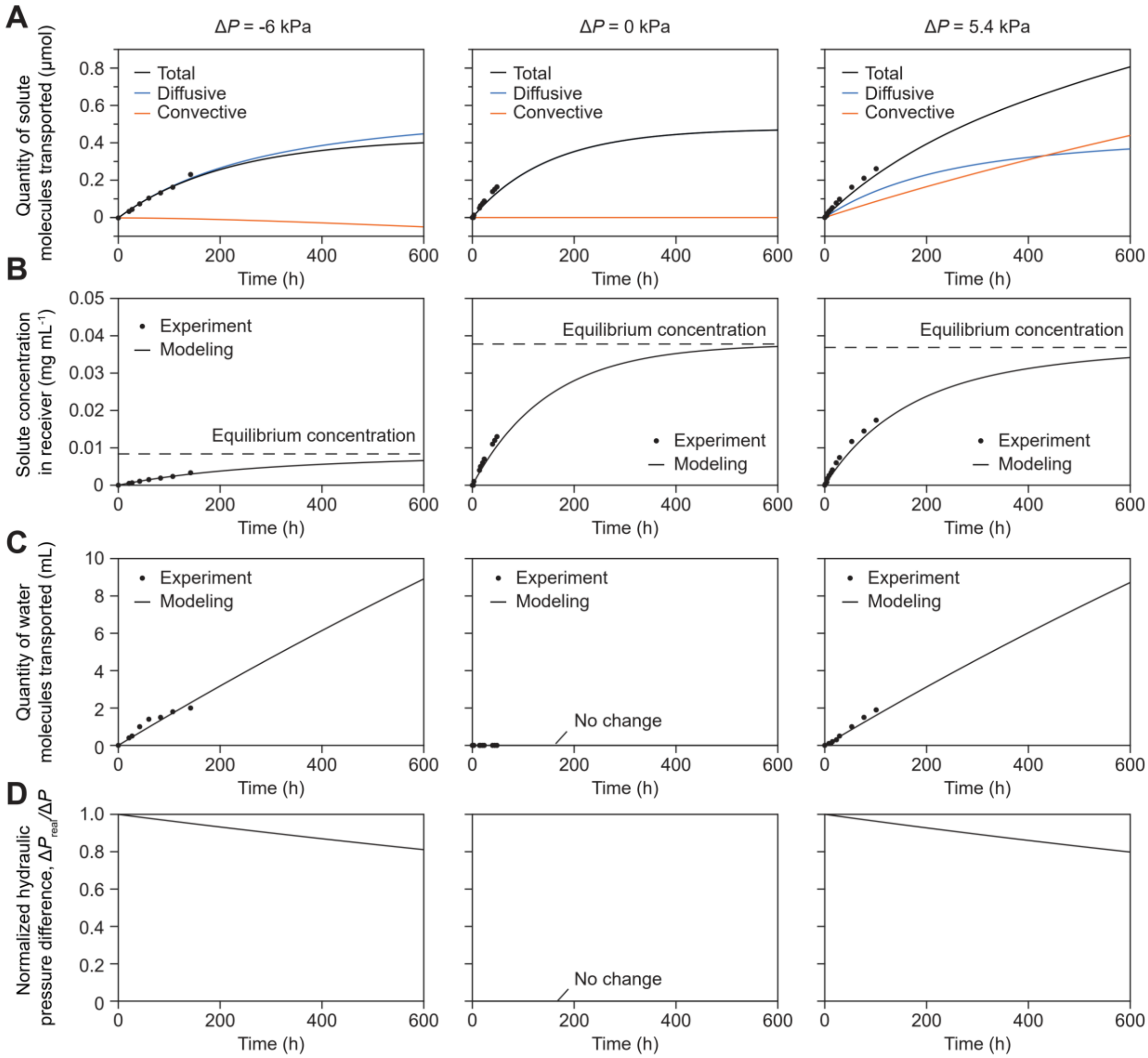

Figure S1. Numerical modeling of diffusion–convection coupled transport across hydrogel membranes. A) Total quantity of solute molecules transported to the receiver chamber under pressure differences of –6, 0, and 5.4 kPa, together with the corresponding diffusive and convective contributions. B) Concentration of solute molecules in the receiver chamber under pressure differences of –6, 0, and 5.4 kPa. C) Total quantity of water molecules transported to the receiver chamber under pressure differences of –6, 0, and 5.4 kPa. D) Normalized hydraulic pressure difference $\Delta P_{real}/\Delta P$ decreases over time due to water redistribution across the hydrogel membrane. Solid dots represent experimental measurements, solid lines represent model predictions, and dashed lines indicate the equilibrium quantities.

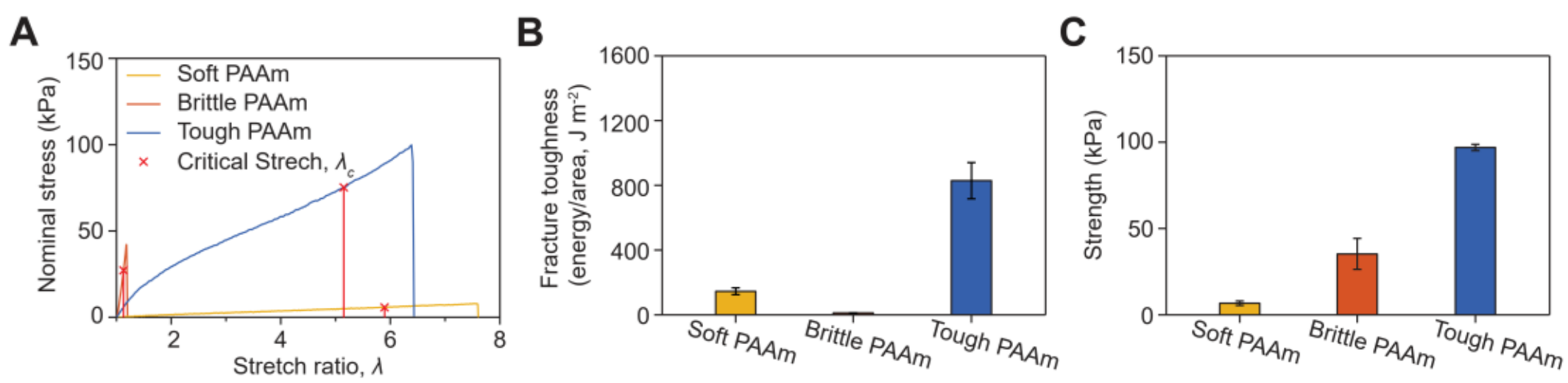

Figure S2. Characterization of fracture toughness of PAAm hydrogels. A) Nominal stress–stretch curves of soft, brittle, and tough PAAm hydrogels obtained from unnotched pure-shear specimens. The critical stretches λ_c from the corresponding notched specimens are indicated. B) Comparison of fracture toughnesses (energy/area) of soft, brittle, and tough PAAm hydrogels. C) Comparison of strength of soft, brittle, and tough PAAm hydrogels.

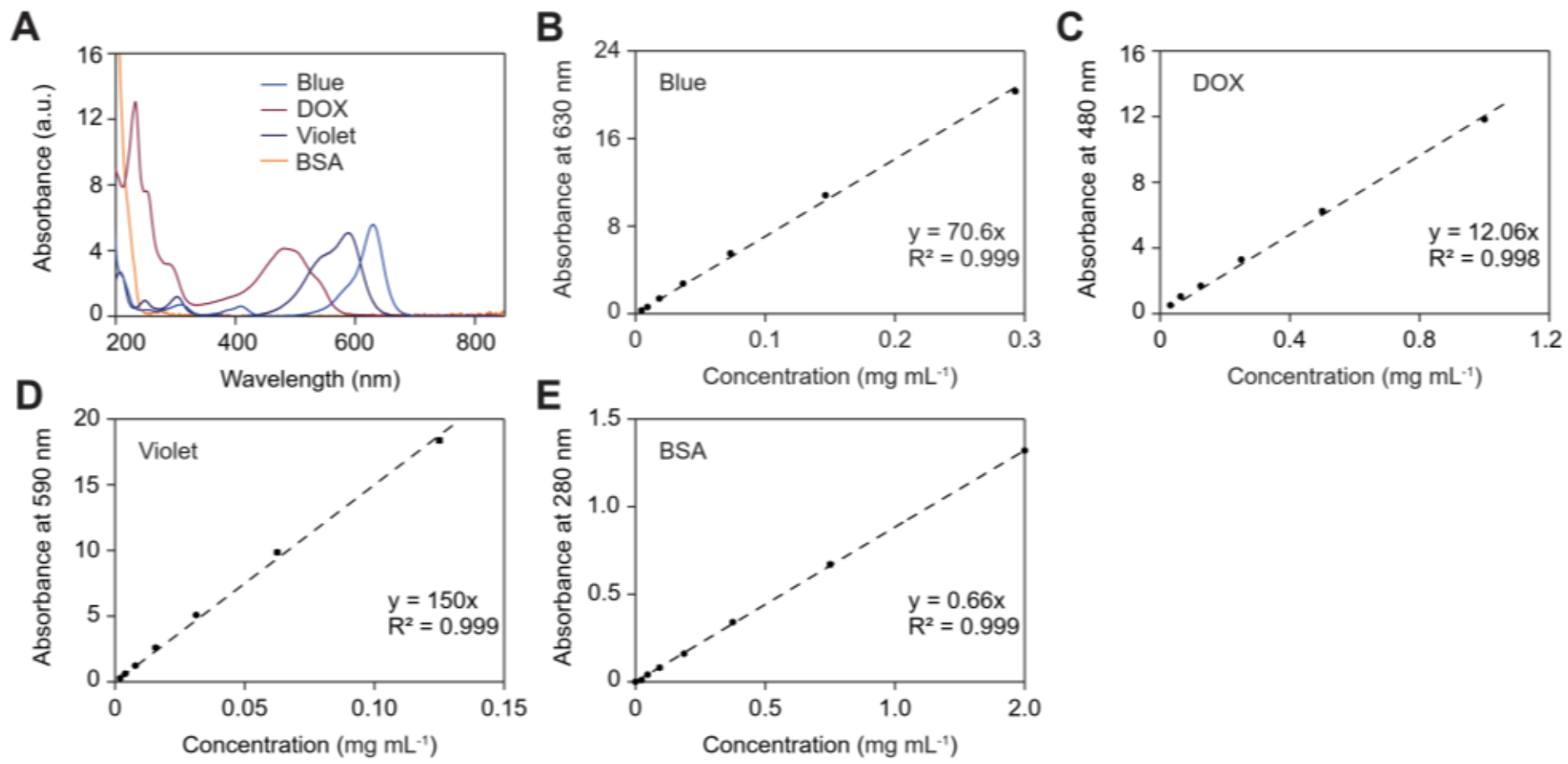

Figure S3. Optical characterization of model solutes. A) UV–Vis absorbance spectra of Blue, DOX, Violet, and BSA from 200 to 850 nm. B-E) Calibration curves relating concentration to absorbance at 630 nm for Blue (B), 480 nm for DOX (C), 590 nm for Violet (D), and 280 nm for BSA (E).

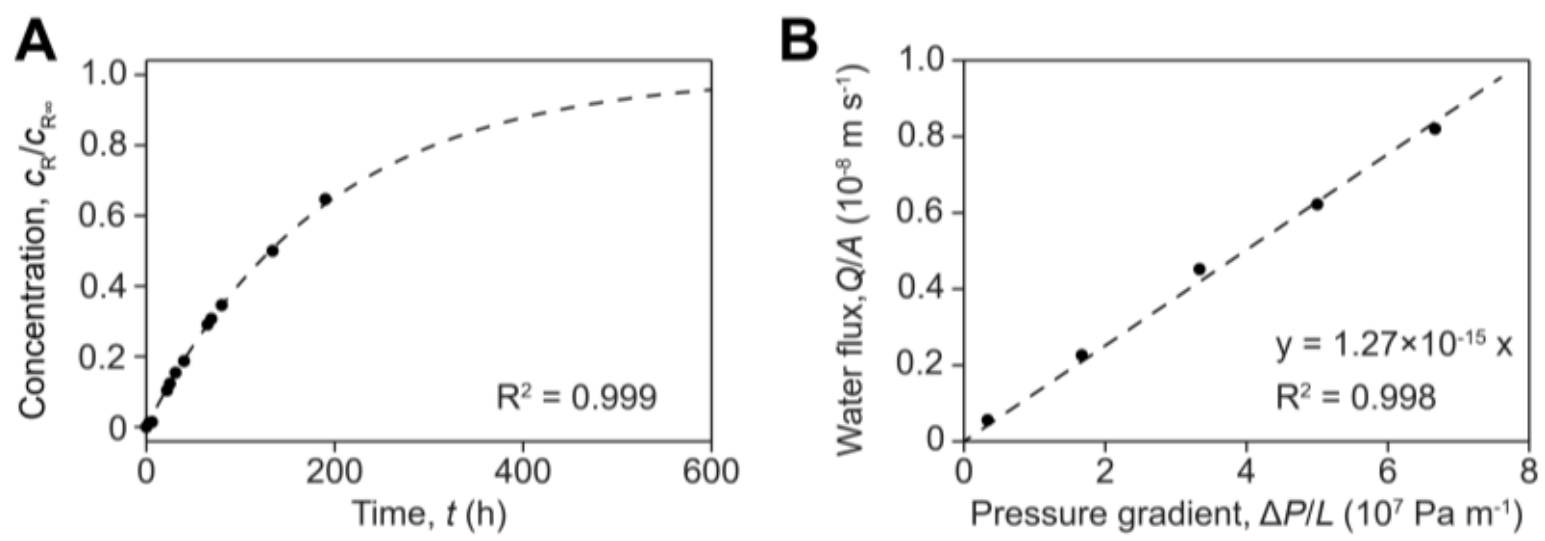

Figure S4. Independent experimental determination of diffusivity and hydraulic permeability. A) Normalized receiver concentration ($c_R/c_{R,\infty}$) as a function of time (t) under the zero-pressure-difference condition. The dashed line represents the fit of the analytical diffusion model to the experimental data, from which the molecular diffusivity (D) was determined. B) Water flux (Q/A) as a function of the applied pressure gradient ($\Delta P/L$). The dashed line represents a linear fit according to Darcy's law, with the slope corresponding to the hydraulic permeability k/μ.

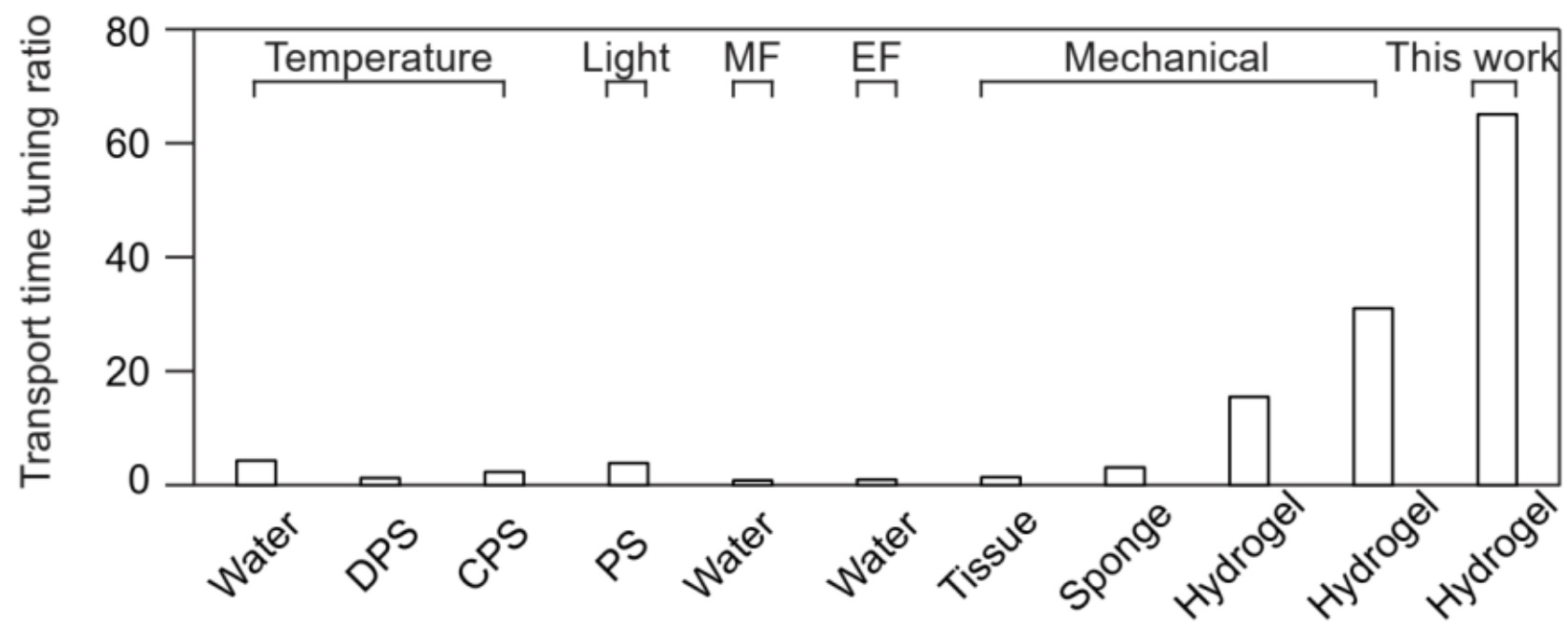

Figure S5. Comparison of tuning ratios for transport timescales across existing approaches and this work. The tuning ratio was defined as the ratio of the maximum transport timescale (τ_{max}) to the minimum transport timescale (τ_{min}). When only diffusivities were reported, the tuning ratio was estimated as the ratio of the minimum diffusivity (D_{min}) to the maximum diffusivity (D_{max}), based on $\tau \sim L^2/D$. Reported transport-regulation approaches include temperature variation from 273 to 333 K in water,(*13*) diluted polymer solutions (DPS),(*14*) and concentrated polymer solutions (CPS),(*15-17*) as well as regulation by light,(*18*) magnetic field (MF),(*19*) electric field (EF),(*20*) and mechanical deformation.(*21-23*) This work achieves the highest tuning ratio through pressure-regulated diffusion–convection coupling.

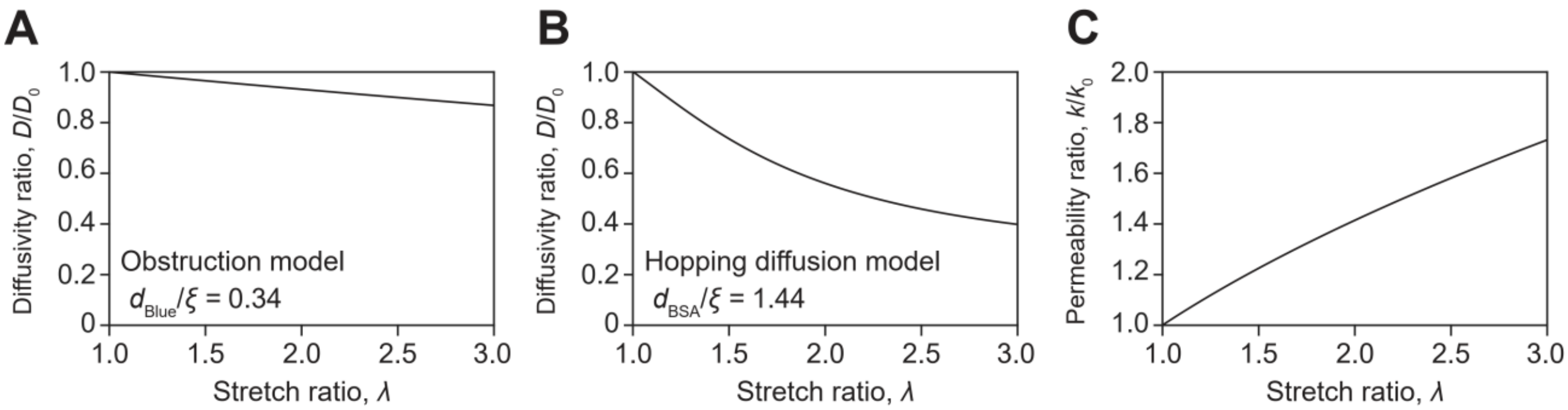

Figure S6. Estimation of deformation-dependent transport parameters in hydrogels. A) Stretch-dependent diffusivity ratio, D/D_0, of the small-molecule solute Blue, predicted using the obstruction model with a solute-to-mesh size ratio of $d_{\mathrm{Blue}}/\xi = 0.34$. B) Stretch-dependent diffusivity ratio, D/D_0, of the large-molecule solute BSA, predicted using the hopping diffusion model with a solute-to-mesh size ratio of $d_{\mathrm{BSA}}/\xi = 1.44$. C) Stretch-dependent permeability ratio, k/k_0, predicted based on the mesh-area-dependent permeability scaling.

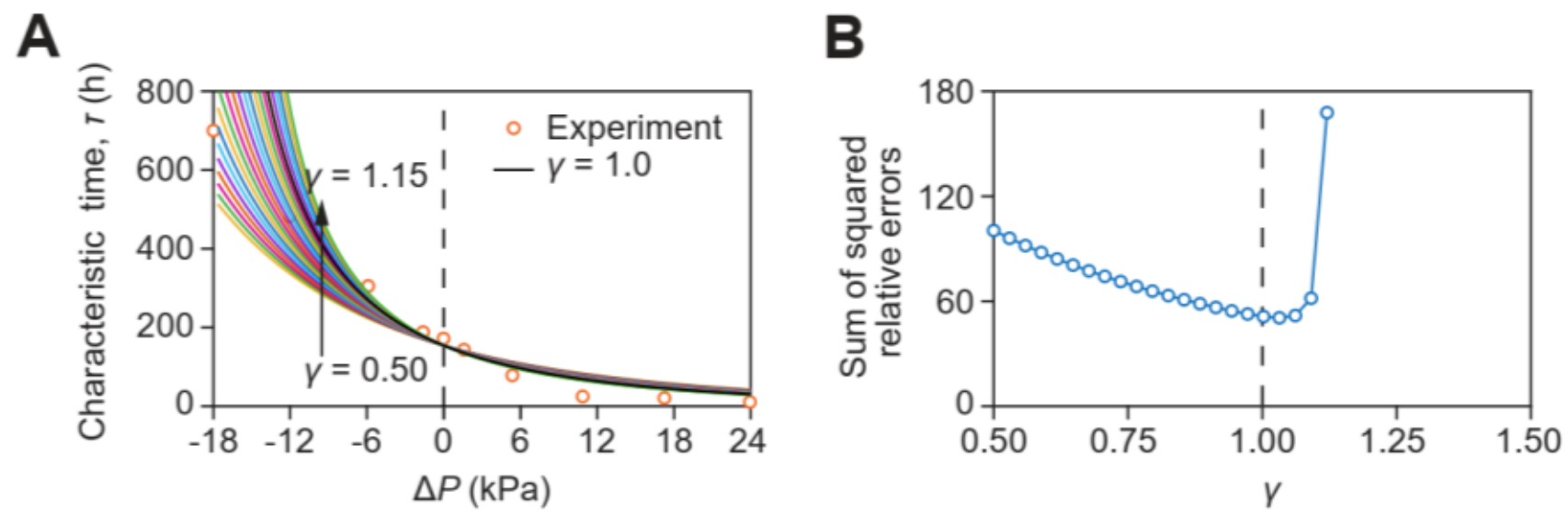

Figure S7. Sensitivity analysis of hydraulic permeability. A) Characteristic time τ as a function of applied pressure difference ΔP. Symbols represent experimental measurements and the solid lines represent fitting using a diffusion–convection coupled model with different permeability values γk (γ = 0.5–1.15). B) Sum of squared relative errors (SSRE) between the model predictions and experimental data with different permeability values γk (γ = 0.5–1.15).

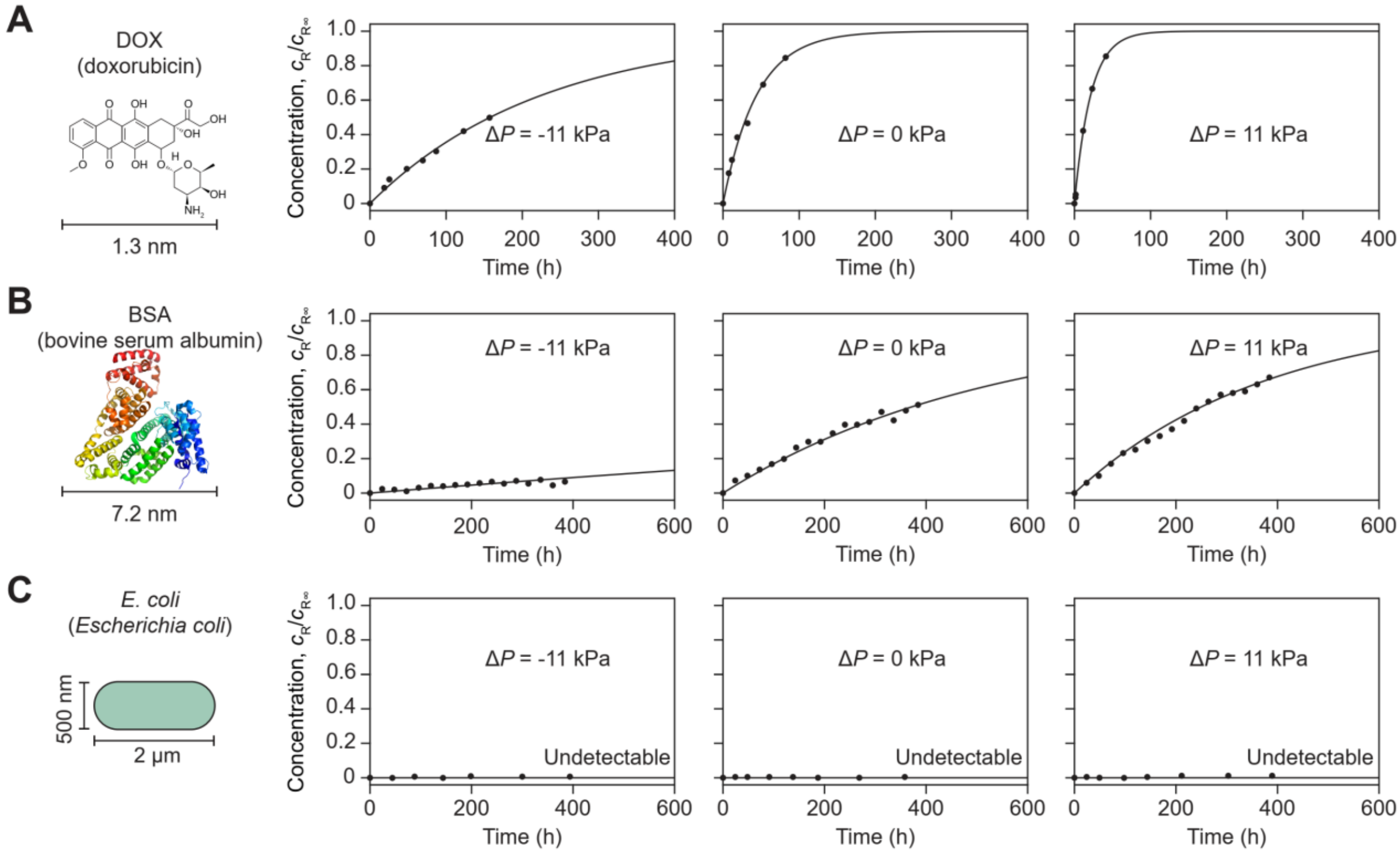

Figure S8. Pressure-regulated transport of DOX, BSA, and *E. coli* across PAAm hydrogel membranes. A) Normalized DOX concentration profiles in the receiver chamber under ΔP = −11, 0, and 11 kPa. B) Normalized BSA concentration profiles in the receiver under ΔP = −11, 0, and 11 kPa. C) Normalized *E. coli* cell density in the receiver under ΔP = −11, 0, and 11 kPa.

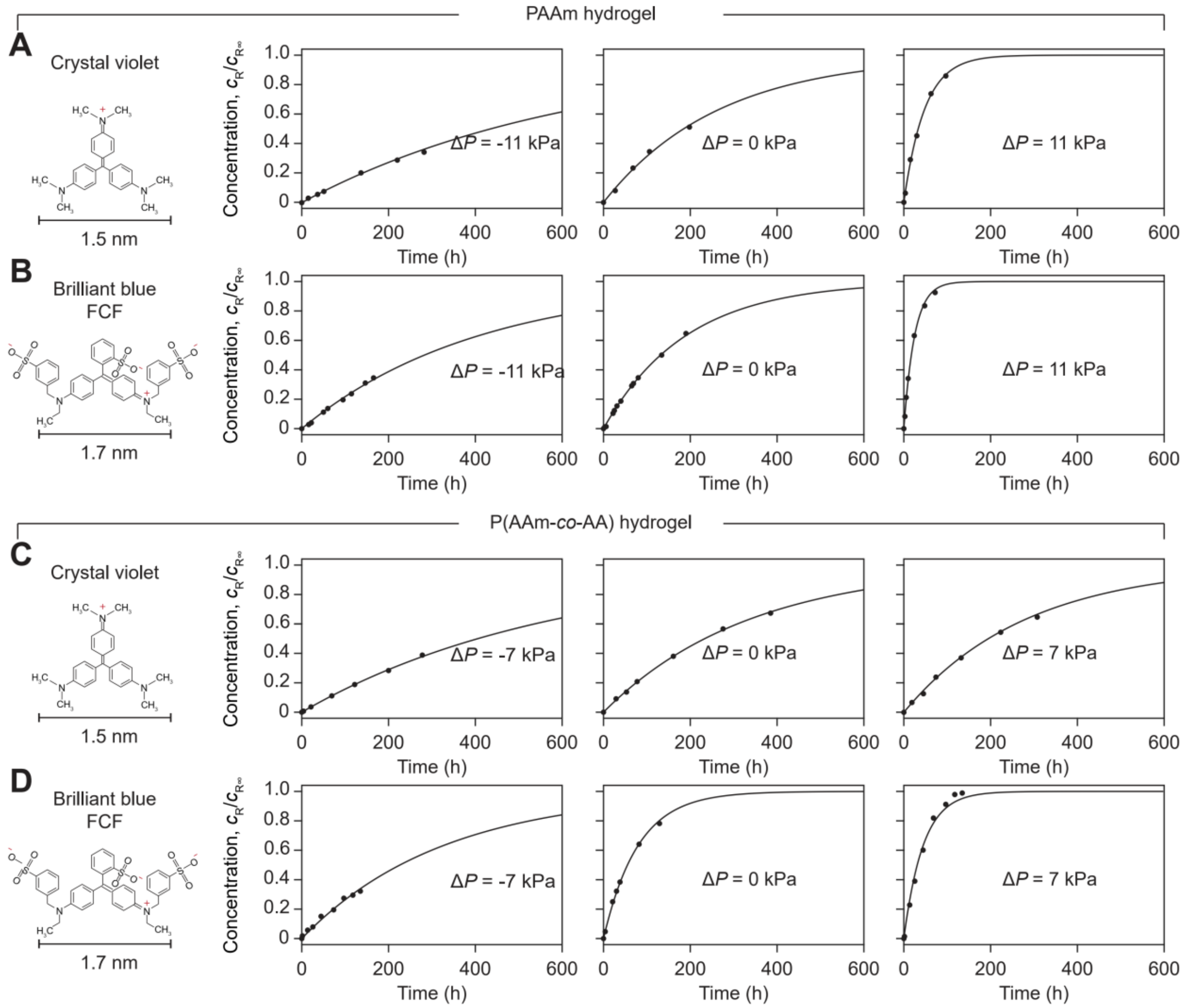

Figure S9. Pressure-regulated transport of Violet and Blue across neutral and charged hydrogel membranes. (A) Normalized Violet concentration profiles in the receiver under $\Delta P = -11$, 0, and 11 kPa. The membrane is a neutral PAAm hydrogel. (B) Normalized Blue concentration profiles in the receiver under $\Delta P = -11$, 0, and 11 kPa. The membrane is a neutral PAAm hydrogel. (C) Normalized Violet concentration profiles in the receiver under $\Delta P = -7$, 0, and 7 kPa. The membrane is a negatively charged P(AAm-*co*-AA) hydrogel. (D) Normalized Blue concentration profiles in the receiver under $\Delta P = -7$, 0, and 7 kPa. The membrane is a negatively charged P(AAm-*co*-AA) hydrogel.

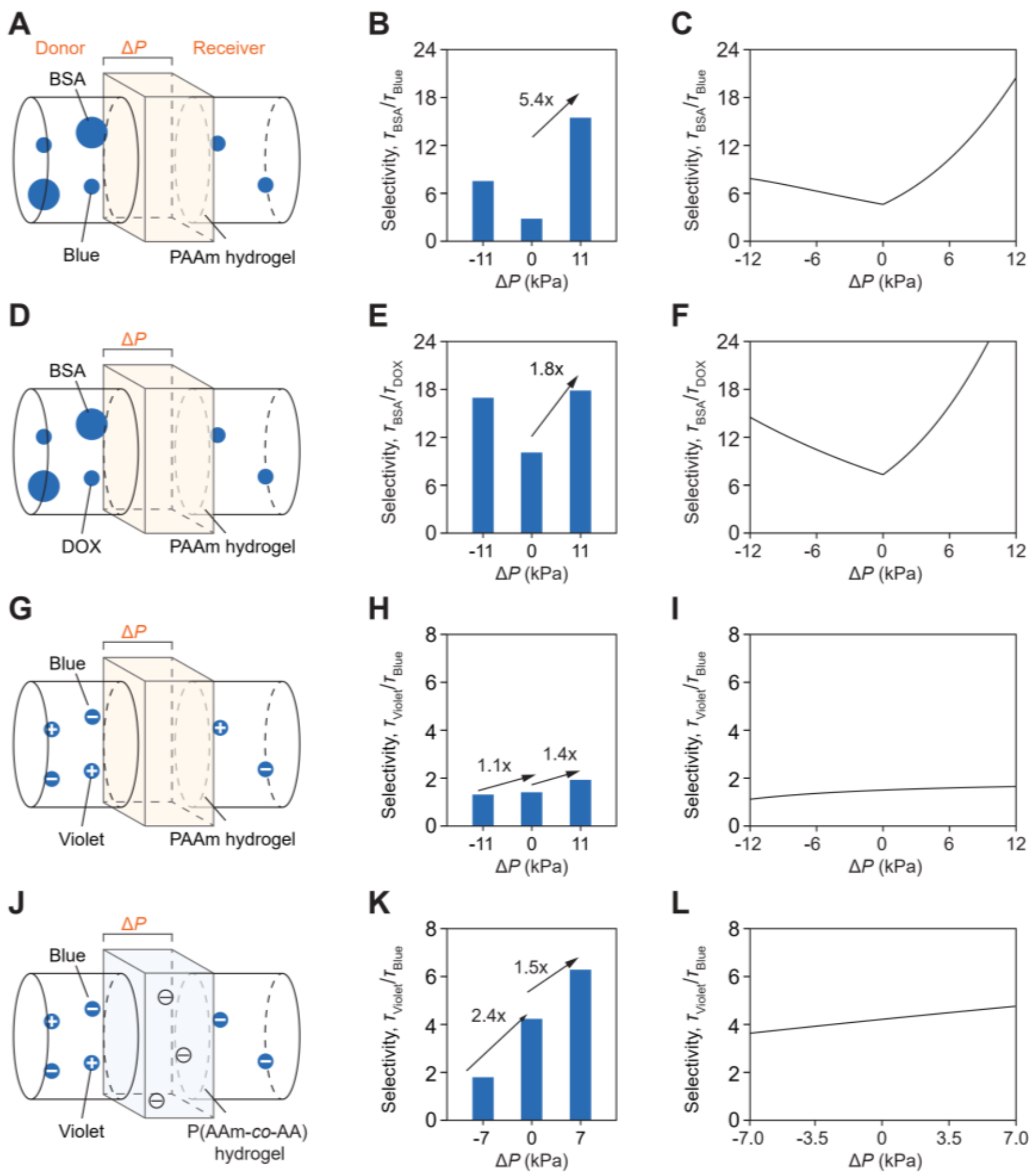

Figure S10. Experimental and simulated pressure-enhanced size- and charge-selectivity across hydrogel membranes. A) Schematic of selective transport between Blue and BSA across a PAAm hydrogel membrane under an applied hydraulic pressure difference. B) Experimental data of transport selectivity between BSA and Blue (τ_{BSA}/τ_{Blue}) as a function of pressure difference ΔP. C) Simulation data for transport selectivity between BSA and Blue (τ_{BSA}/τ_{Blue}) as a function of pressure difference ΔP. D) Schematic of selective transport between DOX and BSA across a PAAm hydrogel membrane under an applied hydraulic pressure difference. E) Experimental data of transport selectivity between BSA and DOX (τ_{BSA}/τ_{DOX}) as a function of pressure difference ΔP. F) Simulation data for transport selectivity between BSA and DOX (τ_{BSA}/τ_{DOX}) as a function of pressure difference ΔP. G) Schematic of selective transport between Blue and Violet across a PAAm hydrogel membrane under an applied hydraulic pressure difference. H) Experimental data of transport selectivity between Violet and Blue ($\tau_{Violet}/\tau_{Blue}$) as a function of pressure difference ΔP. I) Simulation data for transport selectivity between Violet and Blue ($\tau_{Violet}/\tau_{Blue}$) as a function of pressure difference ΔP. J) Schematic of selective transport between Blue and Violet across a P(AAm-*co*-AA) hydrogel membrane under an applied hydraulic pressure difference. K) Experimental data of transport

selectivity between Violet and Blue ($\tau_{\text{Violet}}/\tau_{\text{Blue}}$) as a function of pressure difference ΔP. L) Simulation data for transport selectivity between Violet and Blue ($\tau_{\text{Violet}}/\tau_{\text{Blue}}$) as a function of pressure difference ΔP.

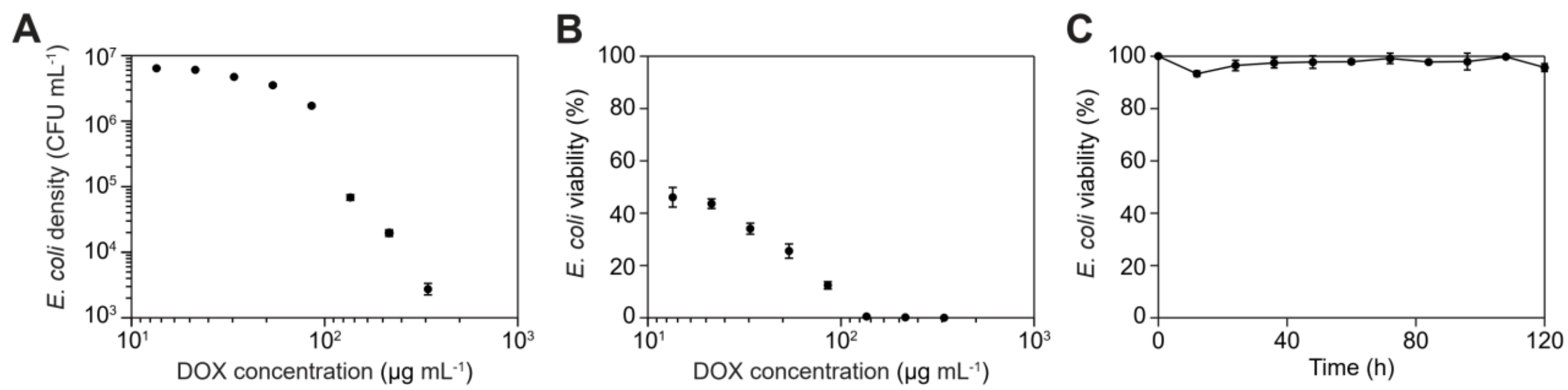

Figure S11. Antimicrobial activity of DOX against *E. coli*. A,B) *E. coli* cell density and viability after treatment with different DOX concentrations. C) *E. coli* viability in PBS at room temperature without any DOX treatment for over five days.

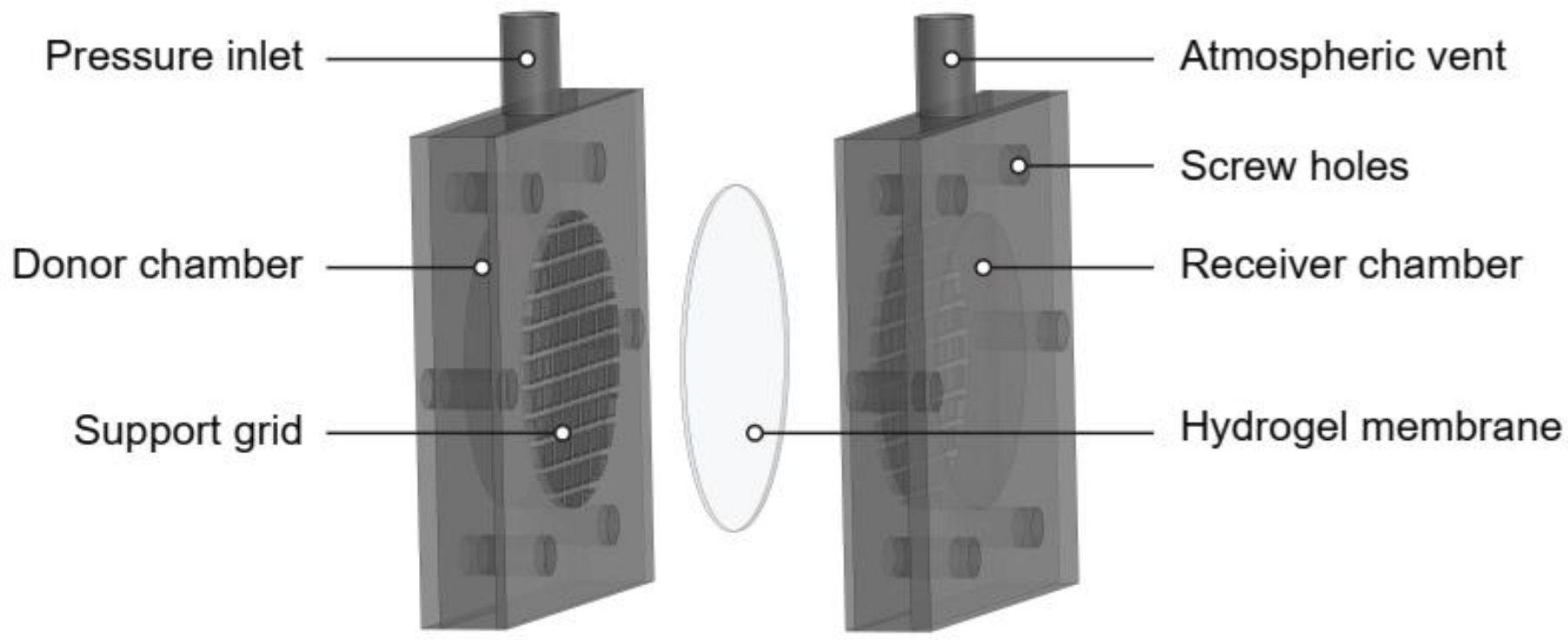

Figure S12. SolidWorks model of 3D-printed plastic H-cell platform. The platform consists of two symmetric halves with monolithically integrated support grids and pressure inlet or atmospheric vent. A fully swollen hydrogel membrane was sandwiched between the two support grids, and two halves were assembled using screws.

Supplementary Tables

Table S1. Comparison of representative studies on interstitial flow and poroelastic transport in soft materials.

Hydrogel materials	Pressure-gradient origin	Configuration	ΔP range	Phenomenon	Independent control of ΔP?	Sustained ΔP loading?	Reference
PNIPAM	Swelling-induced	Free swelling	N/A	Poroelastic solvent redistribution	No	No	(32)
Geltain	Swelling-induced	Free swelling	N/A	Poroelastic solvent redistribution	No	No	(36)
PNIPAM	Swelling-induced	Constrained swelling	N/A	Poroelastic solvent redistribution	No	No	(32)
P(AAm-*co*-SPA)	Swelling-induced	Constrained swelling	N/A	Poroelastic solvent redistribution	No	No	(37)
PEG	Compression-induced	Compression	N/A	Poroelastic solvent redistribution	No	No	(38)
Cellulose	Compression-induced	Compression	2.5 to 3.0 kPa	Poroelastic solvent redistribution	No	No	(39)
Alginate	Compression-induced	Compression	N/A	Poroelastic solvent redistribution	No	No	(40)
Fibrin	Compression-induced	Compression	10 kPa	Interstitial flow	No	No	(41)
PEG	Indentation-induced	Nanoindentation	N/A	Poroelastic solvent redistribution	No	No	(31)
Agarose	Indentation-induced	Indentation	N/A	Poroelastic solvent redistribution	No	No	(42)
Agar	Indentation-induced	Indentation	N/A	Poroelastic solvent redistribution	No	No	(43)
PEG	Indentation-induced	Indentation	N/A	Poroelastic solvent redistribution	No	No	(44)
PAAm	Indentation-induced	Indentation	N/A	Poroelastic solvent redistribution	No	No	(45)
PNIPAM	Indentation-induced	Indentation	N/A	Poroelastic solvent redistribution	No	No	(46)
PDMA	Indentation-induced	Indentation	N/A	Poroelastic solvent redistribution	No	No	(46)
Collagen I	Externally imposed	Microfluidic device	0.1 to 0.5 kPa	Interstitial flow	Yes	No	(47)
Fibrin	Externally imposed	Microfluidic device	0.1 to 0.3 kPa	Interstitial flow	Yes	No	(34)
Collagen I	Externally imposed	Microfluidic device	0.015 kPa	Interstitial flow	Yes	No	(35)
Collagen I	Externally imposed	H-cell	0.1 to 1.0 kPa	Interstitial flow	Yes	No	(48)
Tough PAAm	Externally imposed	H-cell	−18 to +24 kPa	Diffusion–convection coupling	Yes	Yes	This work

Table S2. Synthesis formulations of tough hydrogel, conventional brittle and soft hydrogels.

Compound	Weight fraction (wt.%)		
	**Tough hydrogel**	**Brittle hydrogel**	**Soft hydrogel**
Acrylamide (AAm)	46	14	20
N,*N'*-Methylenebisacrylamide (MBAA)	0.017	5.3	0.002
Ammonium persulfate (APS)	0.008	0.008	0.008
N,N,N',*N'*-Tetramethyl ethylenediamine (TEMED)	0.14	0.14	0.14
Water	54	80	80

Table S3. Summary of hydrodynamic diameters, net charges, molecular weight and specific charge density for all species and hydrogels hydrogel components used in this work.(*24-30*)

Chemicals	Hydrodynamic diameter (nm)	Net charge in PBS (pH = 7.4)	Molecular weight (kDa)	Specific charge density (kDa^{-1})	Reference
Blue	1.68	–2	0.79	–2.52	(24)
DOX	1.35	1	0.54	1.84	(25, 26)
BSA	7.20	–18	66.00	–0.27	(25, 28-30)
Violet	1.51	1	0.41	2.45	(27)
AAm in PAAm	N/A	0	0.07	0.00	This work
AAm:AA = 9:1 molar ratio in P(AAm-*co*-AA)	N/A	–1	0.07	–1.39	This work

References

1. Z. Yu, D. Xu, Z. Usmanova, C. Ye, A. Swarnkar, E. Scott, S. Huang, S. Rao, X. Liu, S. V. Boriskina, "An Elastocaloric Polymer with Ultra-High Solid-State Cooling via Defect Engineering." Advanced Science **13** e18106 (2026).
2. R. S. Rivlin, A. G. Thomas, "Rupture of rubber. I. Characteristic energy for tearing." Journal of Polymer Science **10** 291-318 (1953).
3. R. Long, C.-Y. Hui, "Fracture toughness of hydrogels: measurement and interpretation." Soft Matter **12** 8069-8086 (2016).
4. V. Kapur, J. C. Charkoudian, S. B. Kessler, J. L. Anderson, "Hydrodynamic Permeability of Hydrogels Stabilized within Porous Membranes." Industrial & Engineering Chemistry Research **35** 3179-3185 (1996).
5. Y. Gao, H. J. Cho, "Quantifying the trade-off between stiffness and permeability in hydrogels." Soft Matter **18** 7735-7740 (2022).
6. Y. Cheng, Y. Zhang, Z. Zhao, G. Li, J. Li, A. Li, Y. Xue, B. Zhu, Z. Wu, X. Zhang, "Guanidinium-Decorated Nanostructure for Precision Sonodynamic-Catalytic Therapy of MRSA-Infected Osteomyelitis." Advanced Materials **34** 2206646 (2022).
7. Y. Zhang, Y. Cheng, Z. Zhao, S. Jiang, Y. Zhang, J. Li, S. Huang, W. Wang, Y. Xue, A. Li, "Enhanced chemoradiotherapy for MRSA-infected osteomyelitis using immunomodulatory polymer-reinforced nanotherapeutics." Advanced Materials **36** 2304991 (2024).
8. H. Tyrrell, "The origin and present status of Fick's diffusion law." Journal of chemical education **41** 397 (1964).
9. A. Zannat, A. Lopez, Y. Cheng, J. Ma, M. Delemeester, S. Lin, K. Jayaraman, X. Liu, "Mechanical strain-regulated hydrogel biodegradation for biological scaffolds with programmable lifetime." J Mater Chem B **13** 10485-10499 (2025).
10. B. Amsden, "An obstruction-scaling model for diffusion in homogeneous hydrogels." Macromolecules **32** 874-879 (1999).
11. A. Ogston, "The spaces in a uniform random suspension of fibres." Transactions of the Faraday Society **54** 1754-1757 (1958).
12. J. Liu, S. Lin, "Strain-engineered particle diffusion in uniaxially deformed polymer networks." Journal of the Mechanics and Physics of Solids **191** 105732 (2024).
13. R. C. Reid, T. K. Sherwood, R. E. Street, "The properties of gases and liquids." Physics Today **12** 38-40 (1959).
14. W. W. Graessley, Polymeric liquids & networks: structure and properties, Ed./Eds. Taylor & Francis, 2003), vol. pp.

15. P.-G. De Gennes, Scaling concepts in polymer physics, Ed./Eds. Cornell university press, 1979), vol. pp.
16. P. J. Flory, Principles of polymer chemistry, Ed./Eds. Cornell university press, 1953), vol. pp.
17. D. E. Dunstan, "The viscosity-radius relationship for concentrated polymer solutions." Scientific reports **9** 543 (2019).
18. M. Moniruzzaman, J. M. Bellerby, N. Mai, "The effect of light on the viscosity and molecular mass of nitrocellulose." Polymer degradation and stability **96** 929-935 (2011).
19. S. Ghauri, M. Ansari, "Increase of water viscosity under the influence of magnetic field." Journal of Applied Physics **100** (2006).
20. D. Zong, H. Hu, Y. Duan, Y. Sun, "Viscosity of water under electric field: Anisotropy induced by redistribution of hydrogen bonds." The Journal of Physical Chemistry B **120** 4818-4827 (2016).
21. G. Schwartz, A. Morejon, T. M. Best, A. R. Jackson, F. Travascio, "Strain-dependent diffusivity of small and large molecules in meniscus." Journal of biomechanical engineering **144** 111010 (2022).
22. H. Wu, W.-Z. Fang, Q. Kang, W.-Q. Tao, R. Qiao, "Predicting effective diffusivity of porous media from images by deep learning." Scientific reports **9** 20387 (2019).
23. C. Ye, C. Wei, J. Liu, T. H. Wong, X. Liu, Z. Song, C. Wu, Z. Li, S. Lin, "Mechano-diffusion of particles in stretchable hydrogels." Soft Matter **21** 2230-2241 (2025).
24. I. Roy, A. M. Evans, P. J. Das, M. Ateia, M. R. Ryder, L. O. Jones, M. Kazem-Rostami, S. Goswami, Y. Beldjoudi, D. Shen, G. C. Schatz, J. T. Hupp, W. R. Dichtel, J. F. Stoddart, "Cyclophane-based two-dimensional polymer formed by an interfacial click reaction." Cell Reports Physical Science **3** 100806 (2022).
25. L. Žid, V. Zeleňák, V. Girman, J. Bednarčík, A. Zeleňáková, J. Szűcsová, V. Hornebecq, A. Hudák, M. Šuleková, L. Váhovská, "Doxorobicin as cargo in a redox-responsive drug delivery system capped with water dispersible ZnS nanoparticles." RSC advances **10** 15825-15835 (2020).
26. M. A. Elsawy, J. K. Wychowaniec, L. A. Castillo Díaz, A. M. Smith, A. F. Miller, A. Saiani, "Controlling doxorubicin release from a peptide hydrogel through fine-tuning of drug–peptide fiber interactions." Biomacromolecules **23** 2624-2634 (2022).
27. S. Loera-Serna, E. Ortiz, H. I. Beltrán, "First trial and physicochemical studies on the loading of basic fuchsin, crystal violet and Black Eriochrome T on HKUST-1." New Journal of Chemistry **41** 3097-3105 (2017).
28. D. Devineni, D. Ezekwudo, R. Palaniappan, "Formulation of maltodextrin entrapped in polycaprolactone microparticles for protein and vaccine delivery: Effect of size determining formulation process variables of microparticles on the hydrodynamic diameter of BSA." Journal of Microencapsulation **24** 358-370 (2007).

29. G. Yohannes, S. K. Wiedmer, M. Elomaa, M. Jussila, V. Aseyev, M.-L. Riekkola, "Thermal aggregation of bovine serum albumin studied by asymmetrical flow field-flow fractionation." Analytica Chimica Acta **675** 191-198 (2010).
30. D. Fologea, B. Ledden, D. S. McNabb, J. Li, "Electrical characterization of protein molecules by a solid-state nanopore." Applied physics letters **91** (2007).
31. G. Offeddu, E. Axpe, B. Harley, M. Oyen, "Relationship between permeability and diffusivity in polyethylene glycol hydrogels." AIP advances **8** (2018).
32. J. Yoon, S. Cai, Z. Suo, R. C. Hayward, "Poroelastic swelling kinetics of thin hydrogel layers: comparison of theory and experiment." Soft Matter **6** 6004-6012 (2010).
33. Z.-D. Shi, X.-Y. Ji, H. Qazi, J. M. Tarbell, "Interstitial flow promotes vascular fibroblast, myofibroblast, and smooth muscle cell motility in 3-D collagen I via upregulation of MMP-1." American Journal of Physiology-Heart and Circulatory Physiology **297** H1225-H1234 (2009).
34. S. Zhang, Z. Wan, G. Pavlou, A. X. Zhong, L. Xu, R. D. Kamm, "Interstitial Flow Promotes the Formation of Functional Microvascular Networks In Vitro through Upregulation of Matrix Metalloproteinase-2." Advanced Functional Materials **32** 2206767 (2022).
35. R. Li, J. C. Serrano, H. Xing, T. A. Lee, H. Azizgolshani, M. Zaman, R. D. Kamm, "Interstitial flow promotes macrophage polarization toward an M2 phenotype." Mol Biol Cell **29** 1927-1940 (2018).
36. S. Chen, R. Huang, K. Ravi-Chandar, "Linear and nonlinear poroelastic analysis of swelling and drying behavior of gelatin-based hydrogels." International Journal of Solids and Structures **195** 43-56 (2020).
37. T. Cano, H. Na, J.-Y. Sun, H.-Y. Kim, "Swelling kinetics of constrained hydrogel spheres." Soft Matter **19** 8820-8831 (2023).
38. E. P. Chan, B. Deeyaa, P. M. Johnson, C. M. Stafford, "Poroelastic relaxation of polymer-loaded hydrogels." Soft Matter **8** 8234-8240 (2012).
39. P. Lopez-Sanchez, J. Cersosimo, D. Wang, B. Flanagan, J. R. Stokes, M. J. Gidley, "Poroelastic mechanical effects of hemicelluloses on cellulosic hydrogels under compression." PLoS One **10** e0122132 (2015).
40. S. Cai, Y. Hu, X. Zhao, Z. Suo, "Poroelasticity of a covalently crosslinked alginate hydrogel under compression." Journal of Applied Physics **108** (2010).
41. M. T. Punter, B. E. Vos, B. M. Mulder, G. H. Koenderink, "Poroelasticity of (bio) polymer networks during compression: theory and experiment." Soft matter **16** 1298-1305 (2020).
42. H. Li, X. Lian, D. Guan, "Crossover behavior in stress relaxations of poroelastic and viscoelastic dominant hydrogels." Soft Matter **19** 5443-5451 (2023).
43. D. G. Strange, T. L. Fletcher, K. Tonsomboon, H. Brawn, X. Zhao, M. L. Oyen, "Separating poroviscoelastic deformation mechanisms in hydrogels." Applied Physics Letters **102** (2013).

44. E. P. Chan, Y. Hu, P. M. Johnson, Z. Suo, C. M. Stafford, “Spherical indentation testing of poroelastic relaxations in thin hydrogel layers.” Soft Matter **8** 1492-1498 (2012).
45. Z. I. Kalcioglu, R. Mahmoodian, Y. Hu, Z. Suo, K. J. Van Vliet, “From macro-to microscale poroelastic characterization of polymeric hydrogels via indentation.” Soft Matter **8** 3393-3398 (2012).
46. J. Delavoipiere, Y. Tran, E. Verneuil, A. Chateauminois, “Poroelastic indentation of mechanically confined hydrogel layers.” Soft Matter **12** 8049-8058 (2016).
47. A. Avendano, J. J. Chang, M. G. Cortes-Medina, A. J. Seibel, B. R. Admasu, C. M. Boutelle, A. R. Bushman, A. A. Garg, C. M. DeShetler, S. L. Cole, “Integrated biophysical characterization of fibrillar collagen-based hydrogels.” ACS biomaterials science & engineering **6** 1408-1417 (2020).
48. Z. D. Shi, X. Y. Ji, H. Qazi, J. M. Tarbell, “Interstitial flow promotes vascular fibroblast, myofibroblast, and smooth muscle cell motility in 3-D collagen I via upregulation of MMP-1.” Am J Physiol Heart Circ Physiol **297** H1225-1234 (2009).
